# Enantioselectivity in Vanadium-Dependent Haloperoxidases of Different Marine Sources for Sulfide Oxidation to Sulfoxides

**DOI:** 10.3390/md22090419

**Published:** 2024-09-14

**Authors:** Yun-Han Zhang, Ya-Ting Zou, Yong-Yi Zeng, Lan Liu, Bi-Shuang Chen

**Affiliations:** 1 School of Marine Sciences, Sun Yat-sen University, Zhuhai 519080, China; zhangyh368@mail2.sysu.edu.cn (Y.-H.Z.); zouyt7@mail2.sysu.edu.cn (Y.-T.Z.); zengyy59@mail2.sysu.edu.cn (Y.-Y.Z.); cesllan@mail.sysu.edu.cn (L.L.); 2 Southern Marine Science and Engineering Guangdong Laboratory (Zhuhai), Zhuhai 519080, China

**Keywords:** vanadium haloperoxidase, sulfoxidation, enantioselectivity, oxidation pathway, biocatalysts

## Abstract

This study explores the reasons behind the variations in the enantioselectivity of the sulfoxidation of methyl phenyl sulfide by marine-derived vanadium-dependent haloperoxidases (VHPOs). Twelve new VHPOs of marine organisms were overexpressed, purified, and tested for their ability to oxidize sulfide. Most of these marine enzymes exhibited nonenantioselective behavior, underscoring the uniqueness of *An*VBPO from the brown seaweed *Ascophyllum nodosum* and *Cp*VBPO from the red seaweed *Corallina pilulifera*, which produce (*R*)- and (*S*)-sulfoxides, respectively. The enantioselective sulfoxidation pathway is likely due to direct oxygen transfer within the VHPO active site. This was demonstrated through molecular docking and molecular dynamics simulations, which revealed differences in the positioning of sulfide within *An*VBPO and *Cp*VBPO, thus explaining their distinct enantioselectivities. Nonenantioselective VHPOs probably follow a different oxidation pathway, initiating with sulfide oxidation to form a positively charged radical. Further insights were gained from studying the catalytic effect of VO_4_^3−^ on H_2_O_2_-driven sulfoxidation. This research improves the understanding of VHPO-mediated sulfoxidation and aids in developing biocatalysts for sulfoxide synthesis.

## 1. Introduction

Sulfoxides are crucial in contemporary chemistry due to their widespread presence in functional organic molecules. Their versatility is evident in medicinal chemistry, materials science, and natural product synthesis, where they serve as vital building blocks and sulfur-based ligands [1,2]. From active pharmaceutical ingredients to flavors and fragrances, sulfoxides significantly contribute to everyday products [3,4]. Additionally, they play key roles in catalysis and materials chemistry, fostering innovation in material development and chemical transformations [5]. Consequently, research into synthetic methods for sulfoxides remains active.

The oxidative pathway starting from sulfides is often preferred for synthesizing sulfoxides [6]. Alongside conventional chemical synthesis, biocatalytic approaches are gaining prominence in preparative organic chemistry [7,8]. Notably, mono-oxygenases and peroxidases emerge as appealing enzymes for the enantioselective oxidation of sulfides to sulfoxides. Among mono-oxygenases, Baeyer–Villiger mono-oxygenases, styrene mono-oxygenase, indole mono-oxygenase, and cytochrome P450 mono-oxygenase have been effectively employed in these conversions, each with distinct substrate scopes [9,10,11,12]. Despite the simplicity and efficacy of mono-oxygenase-catalyzed oxidation, they encounter limitations such as the requirement of O_2_ as the oxidant (necessitating intricate electron transport chains) and reliance on the cofactors NAD(P)H for supplying reducing equivalents. Hence, there remains substantial scope for enhancing the practical applications of mono-oxygenases.

New methods involving peroxidases have emerged, facilitating the enantioselective synthesis of sulfoxides by utilizing H_2_O_2_ as both oxygen and electron sources, thereby streamlining the protocols significantly. For instance, heme chloroperoxidase from *Caldariomyces fumago* (*Cfu*CpO) [13,14] and unspecific peroxygenases from *Agrocybe aegerita* (*Aae*UPO) [15], also heme-dependent, have been cited for their ability to synthesize sulfoxides with remarkable enantioselectivities. However, despite the elegance of these approaches, the activity of heme peroxidases can be compromised during catalysis due to the presence of hydrogen peroxide (requiring precise control over H_2_O_2_ supply), thus restricting their applicability in large-scale synthesis.

Vanadium-dependent haloperoxidases (VHPOs), known for their robust tolerance to H_2_O_2_ and organic solvents, emerge as promising biocatalysts for the enantioselective oxidation of sulfides, with notable advancements in this domain. Intriguingly, vanadium-bromoperoxidases from the brown seaweed *Ascophyllum nodosum* (*An*VBPO) and the red seaweed *Corallina pilulifera* (*Cp*VBPO) catalyze the oxidation of methyl phenyl sulfide into (*R*)- and (*S*)-sulfoxides, respectively [16,17]. Conversely, the vanadium-chloroperoxidase from the red seaweed *Curvularia inaequalis* (*Ci*VCPO) produces racemic sulfoxides [16,17,18]. Regarding the mechanistic aspect, prior studies propose that, in *An*VBPO, the aromatic sulfide binds near or within the active site, proceeding through a direct oxygen-transfer step from the peroxo-coordinated enzyme intermediate [16]. In contrast, *Ci*VCPO initiates the catalytic cycle by oxidizing the sulfide to generate a positively charged sulfur radical, which is then liberated from the enzyme and subsequently converted to the sulfoxide through chemical processes [16]. However, conclusive evidence is lacking.

Therefore, several unresolved questions persist: (1) How do other VHPOs from different marine sources catalyze sulfide oxidation? (2) What underlies the opposing enantioselective outcomes of *An*VBPO and *Cp*VBPO despite their structural and sequence similarities? (3) If the proposed mechanism for *Ci*VCPO holds true, can sulfide oxidation be achieved in the presence of Na_3_VO_4_ and H_2_O_2_? Our aim is to provide a comprehensive overview of the enantioselectivities exhibited by different VHPOs in sulfide oxidation reactions and to elucidate the enantioselective and nonenantioselective sulfoxidation mechanisms of VHPOs. This study endeavors to provide scientific insights for the discovery of novel VHPOs for sulfoxide synthesis and to deepen our comprehension of the sulfoxidation mechanism mediated by various VHPOs.

## 2. Results and Discussion

### 2.1. The Enantioselectivity of Sulfoxidation of Methyl Phenyl Sulfide by VHPOs

#### 2.1.1. Discovery of New VHPOs from Different Marine Sources

**Figure 1:**
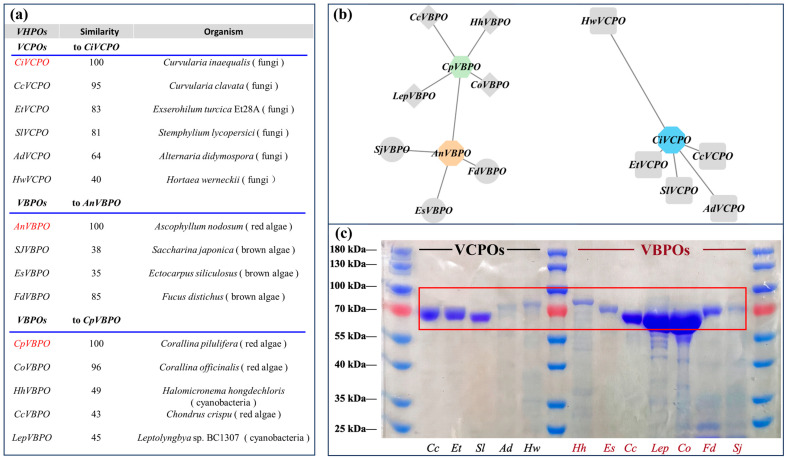
The information of the newly discovered 12 VHPOs: (**a**) VHPOs selected from public databases, revealing their origin and similarity to probe enzymes; (**b**) VHPOs in sequence similarity networks (SSNs); (**c**) SDS-PAGE analysis of the overexpression of the newly discovered VHPOs in *E. coli*.

This study aims to address why marine-derived VHPOs vary in their enantioselectivity when sulfoxidizing methyl phenyl sulfide. To achieve this, we first needed to expand the range of known VHPOs. New VHPOs continue to be discovered from natural sources [19]. For example, thirteen VHPOs have been identified from algae [20], twenty-four from bacteria [21], and three from fungi [19]. However, aside from *An*VBPO, *Cp*VBPO, and *Ci*VCPO, none have been applied to sulfide oxidation. Therefore, we commenced our investigation by overproducing twelve marine-derived putative VHPOs, selected from public databases based on sequence similarity networks (SSNs), using the gene sequences of *Ci*VCPO, *An*VBPO, and *Cp*VBPO as probes to ensure representative diversity (Figure 1, Table S1, and Figures S1–S3).

All of these enzymes were subcloned into pET28a vectors with codon optimization for the heterologous expression in *E. coli*, incorporating an *N*-terminal His_6_-tag. Cultures were initially grown at 37 °C, with overexpression induced at the same temperature using 0.5 mM of IPTG. Despite these efforts, including the use of an autoinduction system [22], the expression levels of the encoded proteins remained low. Fortunately, the monochlorodimedone (MCD) assay allowed us to detect the chloroperoxidase and bromoperoxidase activities of the recombinant proteins [23]. Importantly, all twelve soluble recombinant VHPOs were successfully purified using affinity chromatography and gel filtration (Figure 1). To confirm their catalytic activities, these VHPOs were tested in the Hunsdiecker-type decarboxylative bromination of *p*-coumaric acid (unpublished data), based on previous studies [24].

#### 2.1.2. Sulfoxidation

Next, all twelve obtained VHPOs were tested as catalysts for sulfoxidation. Methyl phenyl sulfide was chosen as the primary test substrate due to its rigid structure and frequent use in selective oxidation reactions. Ten Brink and co-workers reported [16] that *An*VBPO could catalyze the oxidation of methyl phenyl sulfide at concentrations ranging from 0.5 to 20 mM, yielding (*R*)-sulfoxide. Similarly, Zhang’s group described [18] that *Ci*VCPO-mediated sulfoxidation resulted in racemic products with a substrate concentration of 5 mM. Previous studies also showed that the selectivity of sulfoxide production with *An*VBPO increases linearly with enzyme concentrations, but levels off with higher H_2_O_2_ concentrations, achieving the best enantiomeric excess of 93% with 1 μM of enzyme and 10 mM of H_2_O_2_. Consequently, the reaction was conducted at 25 °C with 5 mM of substrate, 1 μM of enzyme, and 10 mM of H_2_O_2_ in sodium acetate buffer (100 mM, pH of 6.0) for 16 h to investigate the catalytic activity of the VHPOs.

**Figure 2:**
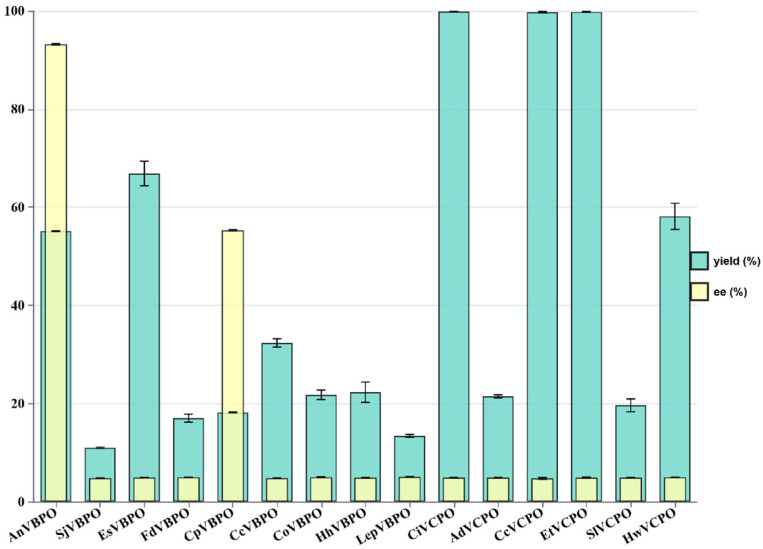
The results of the oxidation of methyl phenyl sulfide catalyzed by newly discovered VHPOs compared to those of *An*VBPO/*Cp*VBPO/*Ci*VCPO. Reaction conditions: [methyl phenyl sulfide] = 5 mM (500 mM stock in acetonitrile), [H_2_O_2_] = 10 mM, [VHPO] = 1 μM in sodium acetate (100 mM, pH of 6.0) for 16 h at 30 °C. Note: for the reaction of *Cc*VCPO and *Et*VCPO, PBS buffer (100 mM, pH of 6.5) was used. *An*VBPO and *Cp*VBPO yielded (*R*)- and (*S*)-products, respectively. The data shown are the results from duplicate experiments. (For reactions exhibiting negligible enantioselectivity, a uniform ee value is adopted in the bar graphs to account for the analytical error associated with liquid chromatography, which is approximately 5%). %ee means enantiomeric excess ($\%ee=\frac{\left[R\right]-[S]}{\left[R\right]+[S]}$ or $\%ee=\frac{\left[S\right]-[R]}{\left[R\right]+[S]}$).

The results, summarized in Figure 2, showed that all of the tested VHPOs catalyzed the oxidation of methyl phenyl sulfide, producing racemic products. Control reactions were conducted simultaneously using thermally inactivated VHPOs or H_2_O_2_ alone, and no reaction occurred in these controls, indicating that the oxidation of methyl phenyl sulfide to sulfoxide was dependent on the active VHPO enzymes. It is reasonable to assume that *Ad*VCPO, *Cc*VCPO, *Et*VCPO, *Sl*VCPO, and *Hw*VCPO share the characteristic of *Ci*VCPO in producing racemic sulfoxide. For other VBPOs, identified using *An*VBPO or *Cp*VBPO as probes, enantioselectivity towards methyl phenyl sulfide was anticipated. However, attempts to achieve enantioselectivity by modifying the reaction conditions (pH, temperature, reaction time, H_2_O_2_ concentration, and enzyme concentration) did not yield any improvement. Thus, the enantioselectivity of the VHPOs from different sources appears to be an inherent property of these enzymes.

### 2.2. The Enantioselective Sulfoxidation Pathway

#### 2.2.1. Labeling Experiment with Labeled Hydrogen Peroxide

**Scheme 1:**
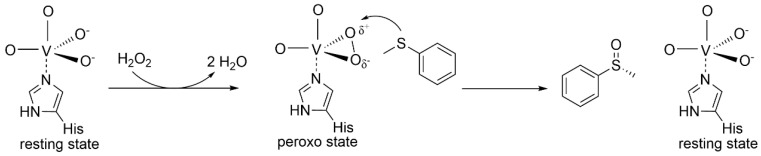
Proposed enantioselective oxidation pathway of direct oxygen transfer to the sulfur atom.

Previously, the oxygen source in methyl phenyl sulfoxide generated by *An*VBPO was explored using ^18^O-labeled hydrogen peroxide [16]. The exclusive formation of ^18^O-labeled sulfoxide suggested that *An*VBPO facilitates the direct and selective transfer of peroxide oxygen to the substrate within the active site (Scheme 1). However, the binding mode of aromatic sulfide in the active site remains elusive, as is the case for *Cp*VBPO. Hence, this section aims to elucidate the enantioselective sulfoxidation mechanism using molecular docking and molecular dynamics (MD) simulation. Initially, molecular docking studies were conducted to pinpoint a potential substrate-binding site for methyl phenyl sulfide (MPS) within the vanadium haloperoxidase protein, utilizing the crystal structure coordinates of vanadate-bound *An*VBPO (PDB entry 1QI9) and *Cp*VBPO (PDB entry 1UP8) [25]. 

#### 2.2.2. Molecular Docking

**Figure 3:**
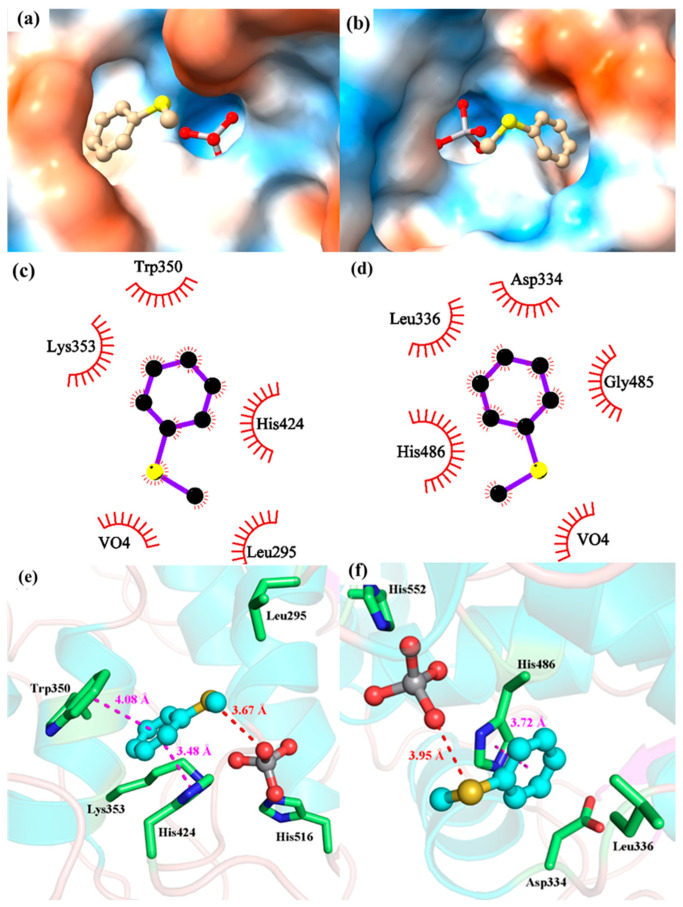
Binding of methyl phenyl sulfoxide to *An*VBPO. (**a**,**b**) The binding of MPS on the hydrophilic and hydrophobic surface of *An*VBPO and *Cp*VBPO, with blue and orange regions representing the hydrophilic and hydrophobic regions of the protein surface, respectively; (**c**,**d**) The two-dimensional binding mode of MPS-*An*VBPO and MPS-*Cp*VBPO, with red gears indicating hydrophobic interactions; (**e**,**f**) The three-dimensional binding mode of MPS-*An*VBPO and MPS-*Cp*VBPO, with purple dashed lines indicating the π-π interaction distance; the red dashed line represents the distance between catalytic atoms.

As shown in Figure 3a,c,e and detailed in Table S2, methyl phenyl sulfide (MPS) is intricately bound within the hydrophobic cavity of the active site of *An*VBPO, positioned close to VO_4_^3−^, thus adopting a favorable catalytic conformation. The hydrophobic cavity primarily consists of Leu295, Trp350, Lys353, and His424. The short distances between Trp350 and His424 and the aromatic sulfide, measured at 4.08 Å and 3.48 Å, respectively, suggest the formation of π-π stacking interactions between the aromatic ring side chains of Trp350 and His424 with MPS. These strong π-π interactions stabilize the benzene ring of MPS, while the methyl sulfur group binds with VO_4_^3−^, creating a favorable catalytic structure. Furthermore, as depicted in Figure 3e, the atomic distance between the sulfur atom in MPS and the oxygen atom in VO_4_^3−^ is 3.67 Å, enhancing the likelihood of catalytic reactions.

Similarly, MPS can bind within the hydrophobic cavity of the active site of *Cp*VBPO, which includes Asp334, Leu336, Gly485, and His486, also positioned near VO_4_^3−^ to adopt a favorable catalytic conformation (see Figure 3b,d,f). Within this hydrophobic cavity, a π-π stacking interaction occurs between the aromatic ring side chains of His486 and the MPS molecule at a distance of 3.72 Å. This strong π-π interaction stabilizes the benzene ring of MPS, while the methyl sulfur group binds with VO_4_^3−^, creating a favorable catalytic structure. As shown in Figure 3f, the atomic distance between the sulfur atom in MPS and the oxygen atom in VO_4_^3−^ is 3.72 Å, indicating a close proximity conductive to catalytic reactions.

**Figure 4:**
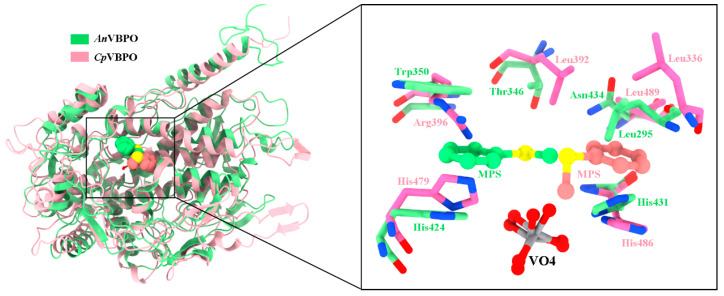
Comparative analysis of the binding of MPS molecules at the active sites of *An*VBPO and *Cp*VBPO.

Molecular docking studies have revealed notable differences in the binding conformations of MPS molecules within *An*VBPO and *Cp*VBPO, potentially leading to the generation of products with distinct enantioselectivities. Experimental findings demonstrate that *An*VBPO catalyzes the oxidation of MPS to yield (*R*)-sulfoxide, while *Cp*VBPO produces (*S*)-sulfoxide. To further investigate the reasons for these divergent product configurations, we examined the binding of MPS within the substrate-binding pockets of the two enzymes. MPS binds to the left and right sides of the VO_4_^3−^ moiety in the substrate-binding pockets of *An*VBPO and *Cp*VBPO, respectively (Figure 4). In *An*VBPO, MPS predominantly occupies the left side, primarily due to the presence of His424 and Trp350 on this side, which form strong π-π stacking interactions with MPS, stabilizing the substrate-binding conformation. Conversely, in *Cp*VBPO, Trp350 is replaced by Arg396, whose relatively flexible side chain impedes the formation of stable interactions with MPS. Consequently, MPS predominantly binds to the right side of *Cp*VBPO, where it engages in π-π stacking interactions with His486. Additionally, numerous hydrophobic amino acids (Leu336, Leu392, and Leu489) above MPS create a hydrophobic environment that stabilizes the benzene ring of MPS. In contrast, in *An*VBPO, these hydrophobic amino acids are replaced by less hydrophobic and more hydrophilic residues (Thr346 and Asn434). Therefore, due to the differences in amino acid distribution near the active site between *An*VBPO and *Cp*VBPO, variations in the binding site between MPS and the proteins result in different configurations of catalytic products.

#### 2.2.3. Molecular Dynamics Simulations

**Figure 5:**
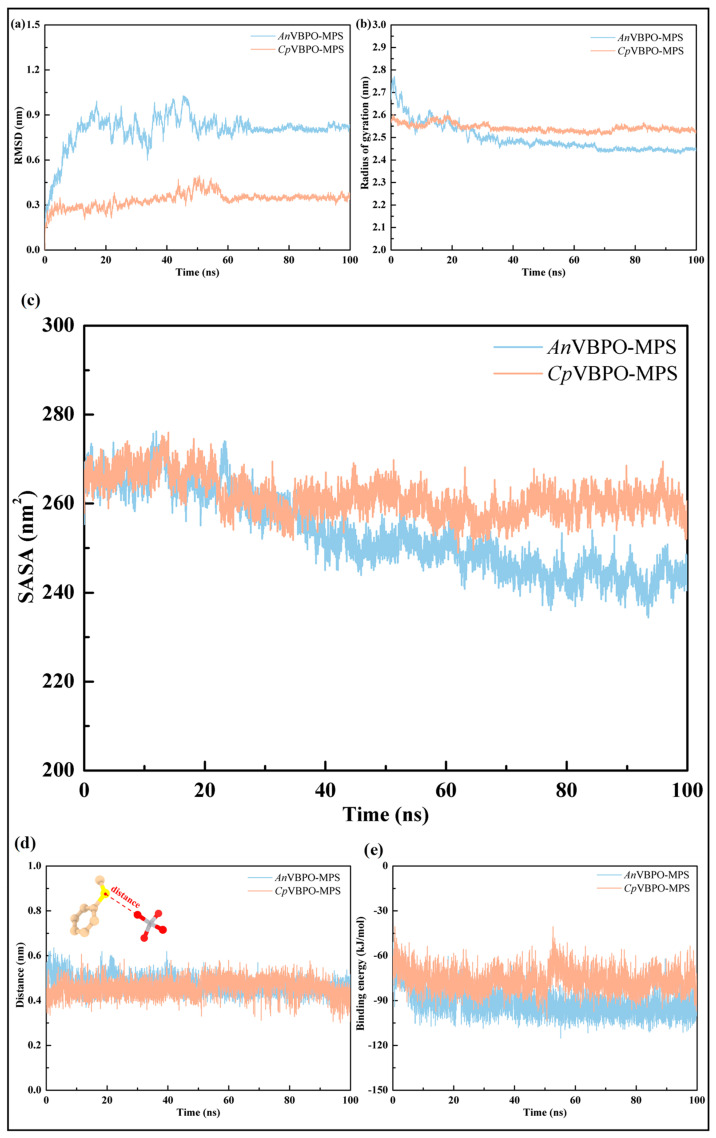
Molecular dynamics (MD) simulations analysis. Backbone RMSD (**a**); Backbone Rg (**b**); Total SASA (**c**); The change in catalytic atomic distance between MPS and VO_4_^3−^ (**d**); The change in binding energy between MPS and the protein (**e**) versus simulation time.

To thoroughly characterize the substrate-binding pocket, we conducted a series of molecular dynamics (MD) simulations involving interactions between *An*VBPO/*Cp*VBPO and the substrate over 100 ns (Figure 5). The Root Mean Square Deviation (RMSD) measures the cumulative atomic deviations between a conformation at a specific time and the target conformation, which is crucial for assessing the system stability. Figure 5a depicts the RMSD variations in both *An*VBPO-MPS and *Cp*VBPO-MPS systems over the simulation time, showing that equilibrium was reached by both systems around 50 ns, with average values of 0.811 ± 0.021 nm and 0.348 ± 0.012 nm, respectively.

The Radius of Gyration (Rg) indicates the compactness of the protein structure [26,27]. As shown in Figure 5b, the Rg value of *An*VBPO initially decreased within the first 40 ns, likely due to a long loop structure in the *N*-terminal region of *An*VBPO. This loop structure exhibited significant motion during the simulation, causing notable changes in both the RMSD and Rg for the entire system. However, the Rg values for *An*VBPO-MPS and *Cp*VBPO-MPS systems tended to stabilize after 60 ns of simulation, with respective values of 2.449 ± 0.009 nm and 2.534 ± 0.010 nm.

This trend is consistent with the behavior of the Total Solvent Accessible Surface Area (SASA), which denotes the surface area of atoms in contact with solvent molecules (Figure 5c) [28,29]. In the initial 50 ns of the MD simulation, both systems exhibited some fluctuations in the SASA values, primarily due to strong interactions between the water molecules surrounding the enzyme and amino acids on the protein surface. As the simulation progressed, interactions between water molecules and the protein gradually stabilized, leading to stabilization of the SASA. On the whole, the average SASA values for *An*VBPO-MPS and *Cp*VBPO-MPS composite systems after 60 ns were 245.01 ± 3.55 nm^2^ and 259.29 ± 2.98 nm^2^, respectively. The RMSD, Rg, and SASA values collectively indicate that both systems reached relatively stable states during the MD simulation.

Additionally, Figure 5d shows that, in both systems, the fluctuations in the catalytic atomic distances between MPS and VO_4_^3−^ are relatively small, with average values of 0.45 ± 0.03 nm and 0.46 ± 0.04 nm after 60 ns, respectively. Overall, the changes in the active sites of both systems during the simulation were minor, indicating that MPS can form stable catalytic structures with both proteins. Figure 5e illustrates that the binding energies between MPS and the proteins in both systems were also relatively stable during the simulation, with only some fluctuations at the initial stage. The average binding energies between MPS and *AnVBPO* and *Cp*VBPO after 60 ns were −95.44 ± 6.39 kJ/mol and −77.60 ± 6.82 kJ/mol, respectively.

**Figure 6:**
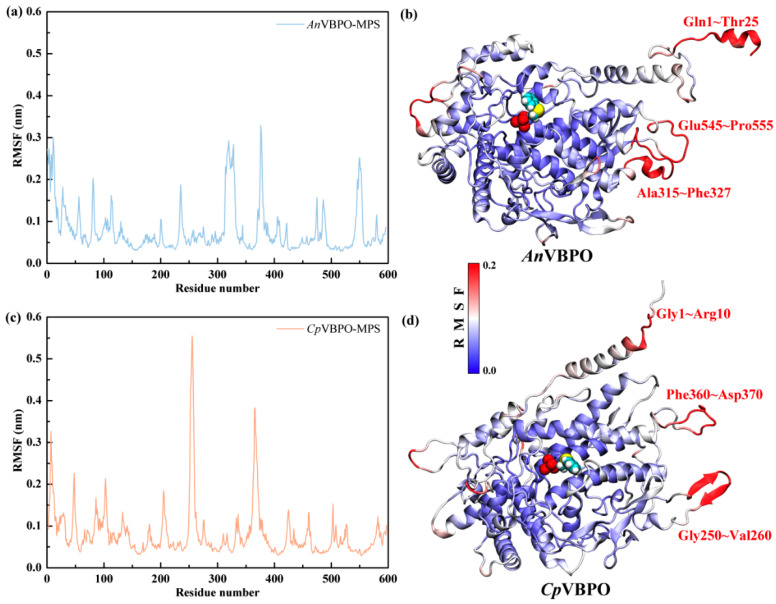
The distribution of root mean square fluctuation (RMSF) for both systems (**a**,**c**) and the corresponding regions exhibiting notable alterations in protein structure (**b**,**d**).

The root mean square fluctuation (RMSF) indicates protein amino acid residue flexibility. Figure 6 shows the amino acid flexibility distribution of *AnVBPO* and *Cp*VBPO throughout the simulation. *An*VBPO generally exhibits low flexibility, with most amino acids below 0.1 nm. Higher flexibility is seen in the regions Gln1~Thr25, Ala315~Phe327, and Glu545~Pro555, mainly in surface loop regions influenced by water solvents. Similarly, *Cp*VBPO shows minor overall flexibility, with most amino acids below 0.1 nm. Regions with greater flexibility are Gly1~Arg10, Gly250~Val260, and Phe360~Asp370, also primarily in surface loop regions affected by water solvents. Thus, the amino acid flexibility near the protein’s active site is low, indicating stable substrate binding and a stable catalytic environment.

### 2.3. The Nonenantioselective Sufoxidation Pathway

**Scheme 2:**
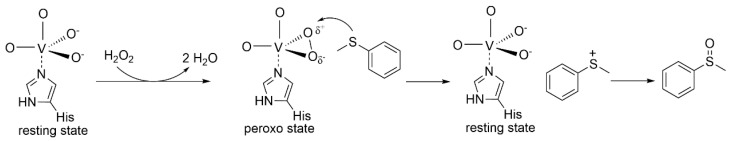
Proposed nonenantioselective oxidation pathway of initiating with sulfide oxidation to form a positively charged radical, which migrates from the enzyme and is subsequently converted to the product via chemical steps.

*An*VBPO and *Cp*VBPO exhibit different enantioselectivities in the oxidation of methyl phenyl sulfoxide, whereas *Ci*VCPO and the newly discovered VHPOs produce racemic products. This suggests a distinct oxidation pathway in *Ci*VCPO and other VHPOs. Previous studies investigating the origin of the oxygen in methyl phenyl sulfoxide produced by *Ci*VCPO using ^18^O-labeled hydrogen peroxide revealed that only 5% of ^18^O was incorporated into the sulfoxide [16]. This finding indicates that *Ci*VCPO follows a different oxidation pathway compared to *An*VBPO and *Cp*VBPO. The authors proposed a mechanism in which the aromatic sulfide undergoes a one-electron transfer step by the peroxointermediate of the enzyme, forming a positively charged sulfur radical that migrates from the enzyme and is subsequently converted to the product through chemical steps (Scheme 2). However, there is no additional evidence to support this mechanism. Currently, we do not have a better method to validate this mechanism, but we are interested in exploring the use of Na_3_VO_4_ and H_2_O_2_ for sulfoxidation. If the combination of Na_3_VO_4_ and H_2_O_2_ can catalyze sulfoxidation, it could provide valuable insights into the mechanism of *Ci*VCPO-catalyzed sulfoxidation.

To test the hypothesis that Na_3_VO_4_ and H_2_O_2_ can catalyze the sulfoxidation, we conducted the oxidation of methyl phenyl sulfoxide (**1a**) by substituting the enzyme with Na_3_VO_4_. Gratifyingly, the desired product, benzyl sulfoxide (**2a**), was significantly detected. After optimizing the reaction conditions, we found that methyl phenyl sulfoxide (**1a**) (5 mM) could be oxidized to **2a** with an 84.4% yield under the catalysis of Na_3_VO_4_ (two equivalents) and H_2_O_2_ (10 mM) at 30 °C in sodium acetate buffer (100 mM, pH of 6.0) for 6 h. It is noteworthy that a series of control experiments using either Na_3_VO_4_ or H_2_O_2_ alone did not promote any sulfoxidation of **1a**, confirming that the sulfoxidation of **1a** was catalyzed by Na_3_VO_4_ in the presence of H_2_O_2_ as a co-substrate. It needs to be emphasized that nonenantioselectivity was observed in the reactions performed with vanadium salt, which supports our assertion that the vanadium salts employ the same mechanism as the non-selective enzymes.

**Figure 7:**
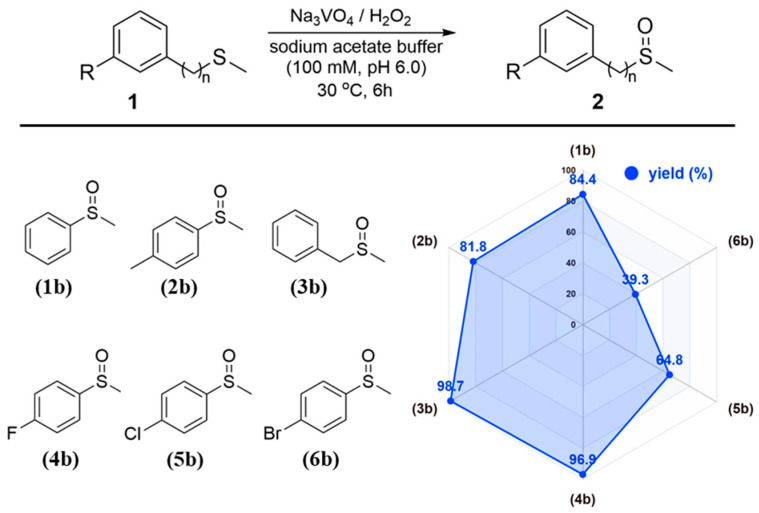
The oxidation of sulfides to sulfoxides with H_2_O_2_ catalyzed by Na_3_VO_4_. Reaction conditions of sulfides to sulfoxides: [sulfide] = 5 mM, [H_2_O_2_] = 10 mM, [Na_3_VO_4_] = 10 mM in 1 mL sodium acetate buffer (100 mM, pH of 6.0), 30 °C for 6 h. Triplicate experiments were performed and the average values are shown. Different product derivatives and their yields are shown.

The study conducted by Zabardasti and Shangaie was recognized for their use of Schiff base vanadium(IV) complexes derived from 2,3-diaminopyridine as catalysts for the efficient oxidation of sulfides to sulfur oxides under similar conditions. Their results emphasized the excellent efficacy of the catalyst, especially in terms of conversion and selectivity [30]. Furthermore, Zhang et al. (2023) synthesized metal–organic frameworks (MOFs) based on poly(vanadium oxide), which exhibited high efficiency in the selective oxidation of sulfides using hydrogen peroxide in methanol at 50 °C. These catalysts not only achieved more than 99% conversion and up to 97% selectivity, but also maintained their structure and catalytic performance after multiple cycles [31]. By comparing our results with those of other groups in the points of substrate concentration, condition reaction, and productivity, it is clear that our system offers a simple and clean route for the preparation of sulfoxides. To elucidate the substrate scope, we tested another five sulfides (**2a**–**6a**) that are structurally similar to methyl phenyl sulfoxide (**1a**) using Na_3_VO_4_ as the catalyst under the previously mentioned reaction conditions. The results are presented in Figure 7. The racemic sulfoxide products, which were commercially available, were used as standards for GC quantification. Notably, all of the tested sulfides were successfully converted into the corresponding sulfoxides by Na_3_VO_4_ and H_2_O_2_. In this section, our aim was to demonstrate whether Na_3_VO_4_ can be used as a catalyst with H_2_O_2_ for sulfoxidation; therefore, we did not apply a wide range of sulfide substrates with structural and electronic diversity. Further characterization and optimization of this promising approach are currently underway in our laboratory.

## 3. Experimental

### 3.1. Materials and Methods

Competent *E. coli* BL21(DE3), *E. coli* Trans 5α, and the vector pET28a(+) were purchased from TransGen Biotech (Beijing, China). All of the chemicals and solvents were purchased from Macklin, Aladdin, or TCI of analytical grade or higher unless otherwise specified. A standard sample of (S)-Methyl phenyl sulfoxide, provided by Sigma-Aldrich (Zhuhai, China), served as the reference. 

The yield of sulfoxides generated from sulfides was analyzed using gas chromatography with a Scion GC 456 system Amsterdam, the Netherlands) equipped with an Agilent J&W GC column (60 m × 0.53 mm × 2.5 µm) (Beijing, China) and nitrogen as the carrier gas. The method was as follows: the temperature profile was 110 °C, holding for 1.2 min; 25 °C/min to 150 °C, holding for 2 min; 30 °C/min to 200 °C for 0.5 min; 30 °C/min to 300 °C for 0.5 min. 

The selectivity of the reactions were determined using an Ultimate Cellu-D(Welch) HPLC column (250 × 4.6 mm, Chiral Technologies) (Beijing, China). After the reaction was completed, the mixture was extracted with an equal volume of ethyl acetate, and then the organic phase was spun to dry, and then redissolved in an equal volume of isopropanol before the subjection to HPLC. Detection method: The detection wavelength was 254 nm. The mobile phase consisted of n-hexane and isopropanol in a ratio of 93:7. The required pump flow rates for the benzyl sulfoxide were as follows: Pump A flow rate was 0.930 mL/min; Pump B flow rate was 0.070 mL/min. The injection volume was 5 µL. 

### 3.2. Gene Synthesis and Subcloning

The genes were codon-optimized for *E. coli*, synthesized, and synthesized and cloned in frame with the *N*-terminal His-tag of the expression vector pET28a(+) between the *Nde*I and *Hind*III restriction sites by Tsingke Biotechnology Co., Ltd. (Beijing, China). 

### 3.3. Heterologous Expression and Purification

Single colonies of the recombinant strains were picked and then incubated in 5 mL of LB medium containing 50 μg/mL kanamycin at 37 °C and 200 rpm for 6 h. The seed broth was transferred to 250 mL of LB medium containing 50 μg/mL of kanamycin with a 2% inoculation dose and shaken at 37 °C and 200 rpm for 3 h. An amount of 0.5 mM of Isopropyl-β-D-thiogalactopyranoside (IPTG) was added to induce the protein expression. After induction at 18 °C for 16 h, the cells were harvested by centrifugation at 4 °C at 12,000× *g* for 20 min. 

The harvested cells expressing enzymes were resuspended in binding buffer (20 mM sodium phosphate, a pH of 7.4, 500 mM of NaCl, 100 μM of sodium orthovanadate, and 20 mM of imidazole), disrupted by ultrasonication in an ice bath, and followed by centrifugation at 12,000× *g* for 20 min to remove the cell debris. The resulting supernatant was loaded onto a Ni-NTA column (5 mL, Sangon Biotech, Shanghai, China) that was pre-equilibrated with buffer A (20 mM of sodium phosphate, a pH of 7.4, 500 mM of NaCl, and 20 mM of imidazole). After washing with washing buffer (20 mM of sodium phosphate, a pH of 7.4, 500 mM of NaCl, and 50 mM of imidazole), the target protein was eluted with elution buffer (w0 mM of sodium phosphate, a pH of 7.4, 500 mM of NaCl, and 250 mM of imidazole). The crude extract was concentrated by an ultrafiltration centrifuge tube (Merck Millipore, 30 kDa or 10 kDa) (Zhuhai, China) and then stored at −20 °C for subsequent analysis. 

### 3.4. General Procedure for Sulfoxidation Catalyzed by VHPOs

Typically, the enzymatic reactions were performed in the following procedures: to a glass vial, **1a** (5 mM, 500 mM of stock in acetonitrile), H_2_O_2_ (10 mM), and 1 μM of enzyme were added into 100 mM of sodium acetate (pH of 6.0). The reaction volume was adjusted to 1 mL and the concentration represented the final concentration of each reaction component. The reaction mixture was incubated in a Thermo shaker (30 °C, 800 rpm) for 16 h. In particular, reactions of *Cc*VCPO and *Et*VCPO were performed using PBS buffer (100 mM, pH of 6.5) with a reaction time of 3.5 h at 35 °C.

### 3.5. Molecular Docking

Molecular docking was conducted using Autodock 4.2.6 from the Scripps Institute in California, USA. Crystal structures of *An*VBPO and *Cp*VBPO were retrieved from the Protein Data Bank (1QI9 for *An*VBPO and 1UP8 for *Cp*VBPO). Water molecules were removed from the crystal structures, which were then employed as receptors for docking. The three-dimensional structure of substrate MPS was generated using ChemDraw (version 16.0). Substrate and VO_4_^3−^ structures were optimized using the ORCA program, and atomic charges were calculated using RESP. Subsequently, Autodock Tools 1.5.6 were utilized to prepare the receptor and substrate structures separately. The docking box was positioned around the enzyme’s active site, near VO_4_^3−^, with center coordinates for *An*VBPO and *Cp*VBPO set at (−14.88, 38.64, −10.61) and (19.26, 67.76, 11.32), respectively. Grid dimensions were set to 40 × 40 × 40 with a grid spacing of 0.375 Å, and 200 molecular docking runs were performed using default parameter values. 

The resulting molecular docking outcomes might exhibit unreasonable atomic contacts and structural strains. To address this, energy optimization was carried out using the Amber14SB force field. The optimization involved the following two steps: first, 2000 steps of the steepest descent method, followed by 2000 steps of the conjugate gradient method to refine the structure further. The optimized structure obtained from this process was used for the subsequent analysis.

### 3.6. Molecular Dynamics Simulation

The two composite structures obtained from molecular docking were placed in simulation boxes for the molecular dynamics (MD) simulations using the Gromacs 2021.5 program. The simulations were conducted under constant temperature, pressure, and periodic boundary conditions, utilizing the Amber14 all-atomic force field and TIP3P water model. In the MD simulation process, hydrogen bonds were constrained using the LINCS algorithm with a time step of 2 fs. Electrostatic interactions were calculated using the Particle Mesh Ewald (PME) method with a non-bonded interaction cutoff of 12 Å updated every 10 steps. The V-rescale temperature coupling method maintained the simulation temperature at 300 K, while the Parrinello–Rahman method controlled the pressure at 1 bar.

Initially, the energy of the four systems was minimized using the steepest descent method to alleviate close atomic contacts. Subsequently, a 100 ps NVT equilibrium simulation at 300 K was performed. Finally, 100 ns MD simulations were conducted for each of the four systems, with conformations saved every 20 ps. Visualization of the simulation results was accomplished using Gromacs embedded programs and VMD.

### 3.7. General Procedure for Sulfoxidation Catalyzed by Na_3_VO_4_ and H_2_O_2_

For the catalytic reaction using sodium orthovanadate as a catalyst, the reaction conditions were as follows: a sodium acetate buffer (100 mM, pH = 6.0) was used, and the concentration of sodium orthovanadate in the solution was 10 mM (which was difficult to dissolve and required ultrasonic heating to dissolve). The reaction volume was adjusted to 1 mL and the concentration represented the final concentration of each reaction component. The reaction mixture was incubated in a Thermo shaker (30 °C, 800 rpm) for 6 h. After the reaction was complete, the mixture was extracted with an equal volume of ethyl acetate, dried over Na_2_SO_4_, and the samples were analyzed by GC. Triplicate experiments were performed. 

### 3.8. General Procedure for Substrate Screening of Na_3_VO_4_ and H_2_O_2_

Reactions were carried out as described in the general procedure for sulfoxidation, using the same concentration (5 mM) for each substrate. After the reaction was complete, the mixture was extracted with an equal volume of ethyl acetate, dried over Na_2_SO_4_, and the samples were analyzed by GC. Triplicate experiments were performed. 

## 4. Conclusions

This study provides significant insights into the enantioselective and nonenantioselective sulfoxidation mechanisms mediated by vanadium-dependent haloperoxidases (VHPOs). Through the overexpression, purification, and testing of twelve new VHPOs, it was found that most of these enzymes exhibit nonenantioselective behavior, underscoring the unique enantioselectivities of *An*VBPO from *Ascophyllum nodosum* and *Cp*VBPO from *Corallina pilulifera*, which produce (*R*)- and (*S*)-sulfoxides, respectively.

Molecular docking and dynamics simulations revealed the distinct positioning of sulfide within *An*VBPO and *Cp*VBPO active sites, explaining their different enantioselectivities. Nonenantioselective VHPOs likely follow a different oxidation pathway, starting with the formation of a positively charged radical. Further studies demonstrated that Na_3_VO_4_ and H_2_O_2_ can catalyze sulfoxidation, supporting the proposed mechanism for nonenantioselective VHPOs like *Ci*VCPO. The labeling experiment confirmed that the oxygen source in *Ci*VCPO-catalyzed sulfoxidation differs from that in *An*VBPO and *Cp*VBPO, indicating a distinct oxidation pathway.

Overall, this research enhances understanding of VHPO-mediated sulfoxidation, providing valuable insights for developing biocatalysts for sulfoxide synthesis. It emphasizes the inherent properties of VHPOs from different sources and their potential applications in synthetic organic chemistry.

## Data Availability

The original data presented in the study are included in the article/Supplementary Material; further inquiries can be directed to the corresponding author.

## Supplementary Materials

The following supporting information can be downloaded at: https://www.mdpi.com/article/10.3390/md22090419/s1. Figure S1: Protein sequence alignment of putative VHPOs with *An*VBPO, *Cp*VBPO, and *Ci*VCPO; Figure S2–S18: GC chromatograms and chiral HPLC chromatograms for all the substrates and the corresponding products mentioned in this study. Table S1: The amino acid sequences of all the vanadium-dependent haloperoxidases (VHPOs) used in this study; Table S2: Molecular docking results of MPS molecular with *An*VBPO and *Cp*VBPO.

## References

[1] Fu Z.H., Tian H.D., Ni S.F., Wright J.S., Li M., Wen L.R., Zhang L.B. (2022). Scalable selective electrochemical oxidation of sulfides to sulfoxides. Green Chem..

[2] Park J.K., Lee S. (2021). Sulfoxide and sulfone synthesis via electrochemical oxidation of sulfides. J. Org. Chem..

[3] Liu M., Shi S., Zhao L., Wang M., Zhu G., Zheng X., Gao J., Xu J. (2018). Effective utilization of in situ generated hydroperoxide by a Co–SiO_2_@Ti–Si core–shell catalyst in the oxidation reactions. ACS Catal..

[4] Han J., Soloshonok V.A., Klika K.D., Drabowicz J., Wzorek A. (2018). Chiral sulfoxides: Advances in asymmetric synthesis and problems with the accurate determination of the stereochemical outcome. Chem. Soc. Rev..

[5] Wojaczyńska E., Wojaczyński J. (2020). Modern stereoselective synthesis of chiral sulfinyl compounds. Chem. Rev..

[6] Liu K., Meng J., Jiang X. (2023). Gram-scale synthesis of sulfoxides via oxygen enabled by Fe(NO_3_)_3_·9H_2_O. Org. Process Res. Dev..

[7] Fan C., Xie Z., Zheng D., Zhang R., Li Y., Shi J., Cheng M., Wang Y., Zhou Y., Zhan Y. (2024). Overview of indigo biosynthesis by flavin-containing monooxygenases: History, industrialization challenges, and strategies. Biotechnol. Adv..

[8] Paul C.E., Eggerichs D., Westphal A.H., Tischler D., van Berkel W.J.H. (2021). Flavoprotein monooxygenases: Versatile biocatalysts. Biotechnol. Adv..

[9] Goundry W.R.F., Adams B., Benson H., Demeritt J., McKown S., Mulholland K., Robertson A., Siedlecki P., Tomlin P., Vare K. (2017). Development and scale-up of a biocatalytic process to form a chiral sulfoxide. Org. Process Res. Dev..

[10] Nikodinovic-Runic J., Coulombel L., Francuski D., Sharma N.D., Boyd D.R., Ferrall R.M.O., O’Connor K.E. (2013). The oxidation of alkylaryl sulfides and benzobthiophenes by *Escherichia coli* cells expressing wild-type and engineered styrene monooxygenase from Pseudomonas putida CA-3. Appl. Microbiol. Biotechnol..

[11] Willrodt C., Gröning J.A.D., Nerke P., Koch R., Scholtissek A., Heine T., Schmid A., Bühler B., Tischler D. (2020). Highly efficient access to (*S*)-Sulfoxides utilizing a promiscuous flavoprotein monooxygenase in a whole-cell biocatalyst format. ChemCatChem.

[12] Schallmey A., den Besten G., Teune I.G.P., Kembaren R.F., Janssen D.B. (2011). Characterization of cytochrome P450 monooxygenase CYP154H1 from the thermophilic soil bacterium *Thermobifida fusca*. Appl. Microbiol. Biotechnol..

[13] Bormann S., Baraibar A.G., Ni Y., Holtmann D., Hollmann F. (2015). SpeCific oxyfunctionalisations catalysed by peroxygenases: Opportunities, challenges and solutions. Catal. Sci. Technol..

[14] Bassanini I., Ferrandi E.E., Vanoni M., Ottolina G., Riva S., Crotti M., Brenna E., Monti D. (2017). Peroxygenase-catalyzed enantioselective sulfoxidations. Eur. J. Org. Chem..

[15] Sang X., Tong F., Zeng Z., Wu M., Yuan B., Sun Z., Sheng X., Qu G., Alcalde M., Hollmann F. (2022). A biocatalytic platform for the synthesis of enantiopure propargylic alcohols and amines. Org. Lett..

[16] ten Brink H., Schoemaker H.E., Wever R. (2001). Sulfoxidation mechanism of vanadium bromoperoxidase from *Ascophyllum nodosum*. Eur. J. Biochem..

[17] Brink H.B.T., Tuynman A., Dekker H.L., Hemrika W., Izumi Y., Oshiro T., Schoemaker H.E., Wever R. (1998). Enantioselective sulfoxidation catalyzed by vanadium haloperoxidases. Inorg. Chem..

[18] Wang P., Han X., Liu X., Lin R., Chen Y., Sun Z., Zhang W. (2022). Synthesis of enantioenriched sulfoxides by an oxidation- reduction enzymatic cascade. Chem. Eur. J..

[19] Cochereau B., Le Strat Y., Ji Q., Pawtowski A., Delage L., Weill A., Mazéas L., Hervé C., Burgaud G., Gunde-Cimerman N. (2023). Heterologous expression and biochemical characterization of a new chloroperoxidase isolated from the deep-sea hydrothermal vent black yeast *Hortaea werneckii* UBOCC-A-208029. Mar. Biotechnol..

[20] Wischang D., Radlow M., Schulz H. (2012). Molecular cloning, structure, and reactivity of the second bromoperoxidase from *Ascophyllum nodosum*. Bioorg. Chem..

[21] Mckinnie S.M.K., Miles Z.D., Moore B.S. (2018). Characterization and biochemical assays of streptomyces vanadium dependent chloroperoxidases. Methods Enzymol..

[22] Studier F.W. (2005). Protein production by auto-induction in high density shaking cultures. Protein Expr. Purif..

[23] Wever R., Barnett P. (2017). Vanadium chloroperoxidases: The missing link in the formation of chlorinated compounds and chloroform in the terrestrial environment?. Chem. Asian J..

[24] Li H., Younes S.H.H., Chen S., Duan P., Cui C., Wever R., Zhang W., Hollmann F. (2022). Chemoenzymatic Hunsdiecker-type decarboxylative bromination of cinnamic acids. ACS Catal..

[25] Tao X., Huang Y., Wang C., Chen F., Yang L., Ling L., Che Z., Chen X. (2020). Recent developments in molecular docking technology applied in food science: A review. Int. J. Food Sci. Tech..

[26] Mirete S., Morgante V., Gonźalez-Pastor J.E. (2016). Functional metagenomics of extreme environments. Curr. Opin. Biotechnol..

[27] Ferrer M., Golyshina O., Beloqui A., Golyshin P.N. (2007). Mining enzymes from extreme environments. Curr. Opin. Microbiol..

[28] Parra-Cruz R., Jäger C.M., Lau P.L., Gomes R.L., Pordea A. (2018). Rational design of thermostable carbonic anhydrase mutants using molecular dynamics simulations. J. Phys. Chem. B.

[29] Du J., Dong J., Du S., Zhang K., Yu J., Hu S., Yin H. (2020). Understanding thermostability factors of barley limit dextrinase by molecular dynamics simulations. Front. Mol. Biosci..

[30] Zabardasti A., Shangaie S.A. (2019). Vanadium (IV) complexes with Schiff base ligands derived from 2, 3-diaminopyridine as catalyst for the oxidation of sulfides to sulfoxides with H_2_O_2_. J. Iran. Chem. Soc..

[31] Zhang J.L., Zhao Q.X., Cheng M.Y., Xuan W.M., Liu Y. (2023). Polyoxovanadate-based metal–organic frameworks consisted of open vanadium sites for selective catalytic oxidation of sulfides. Tungsten.

