# Supplementary material for: Enantioselectivity in Vanadium-Dependent Haloperoxidases of Different Marine Sources for Sulfide Oxidation to Sulfoxides
Source: Mar Drugs. 2024 Sep 14;22(9):419. doi: 10.3390/md22090419 (PMC11433219; doi:10.3390/md22090419)
Supplement: Supplementary file 1 [file marinedrugs-22-00419-s001.zip › marinedrugs-3190975-supplementary.pdf]

Supporting Information

Enantioselectivity in Vanadium-Dependent Haloperoxidases of Different Marine Sources for Sulfide Oxidation to Sulfoxides

Yun-Han Zhang[1], Ya-Ting Zou[1], Yong-Yi Zeng[1], Lan Liu[1,2], Bi-Shuang Chen[1,2]*

1. School of Marine Sciences, Sun Yat-Sen University, Zhuhai 519080, China.
2. Southern Marine Science and Engineering Guangdong Laboratory (Zhuhai), Zhuhai 519080, China.

Corresponding Author:

Bi-Shuang Chen: chenbsh23@mail.sysu.edu.cn / (+86) 0756-3668775

Table S1. Sequence Information

Enzyme	Originism	Sequence
CiVCPO	*Curvularia inaequalis*	MGSVTPIPLPKIDEPEEYNTNYILFWNHVGLELNRVTHTVGGPLTGPPLSAR<br>ALGMLHLAIHDAYFSICPPTDFTTFLSPDTENAAYRLPSPNGANDARQAVA<br>GAALKMLSSLYMKPVEQPNPNPGANISDNAYAQLGLVLDRSVLEAPGGV<br>DRESASFMFGEDVADVFFALLNDPRGASQEGYHPTPGRYKFDDEPTHPVV<br>LIPVDPNNPNGPKMPFRQYHAPFYGKTTKRFATQSEHFLADPPGLRSNAD<br>ETAEYDDAVRVAIAMGGAQALNSTKRSPWQTAQGLYWAYDGSNLIGTPP<br>RFYNQIVRRIAVTYKKEEDLANSEVNNADFARLFALVDVACTDAGIFSWKE<br>KWEFEFWRPLSGVRDDGRPDHGDPFWLTLGAPATNTNDIPFKPPFPAYPS<br>GHATFGGAVFQMVRRYYNGRVGTWKDDEPDNIAIDMMISEELNGVNRD<br>LRQPYDPTAPIEDQPGIVRTRIVRHFDSAWELMFENAISRIFLGVHWRFDA<br>AAARDILIPTTTKDVYAVDNNGATVFQNVEDIRYTTRGTREDPEGLFPIGGV<br>PLGIEIADEIFNNGLKPTPPEIQPMPQETPVQKPVGQQPVKGMWEEEQAP<br>VVKEAP
HwVCPO	*Hortaea*	MIPLHQRPEDTDYNKNYVLYWNHVALELVRLTHTETASGAVNGPPLVAR

	werneckii	MLGILHLAIHDAFFALHNTAGISTYLSPTQSAPYRLPDILDARDGRQAVAGA<br>AITVLEDQYFVSHPSESFSANDQAEQLLRRYITAFAPDTLSSSYRFGYEVGKA<br>MLKLLAIKQDEPGTDQDSYMPSQGQYRFFHDPTNPVVVSSVDPNDPESPK<br>RASRVAHAPFYGMTAKRLAVQYRIGKDQTEHIIADPPVGFGEADMPEYVD<br>ALRDVYRMGGRTELNTTTRRPWQTAAAHFWAYDGSNLIGVPLRLYNQILR<br>TVAWDHRPDKRIPDSDKNNVEFARLFALCNAAMADAGIFAWQEKYCFEF<br>WRPLSGVREASTDLIVRNRGNGMVEGIPDPDRPPTMDDGTVALQDPFW<br>LELGAPNTNSNRIPFKPAFPAYPSGHATFGAACFQMMRLYYKNSHRDGIA<br>DFDIDGPDNIAFDFVSDEQDGRNRDNLRNPFDPNVPIDDQPGIVRALWKR<br>RFGSLWEALWENAISRVWLGVHWRFDAFSPKDALVRNPTPTTVSSKTDSP<br>LLYAVETDHSTRYKPLAEVRYTSTGTRRDRPGKNFPVGGVPLGIGIANDIFQ<br>GGLRPTAQNKQPSGRHRCGRLGKP
AdVCPO	*Alternaria didymospora*	MTIDFTPVELPVVEEDAEYNWNYILFWNNVGLELNRVTHTFGALKAGPPL<br>SPRALGMLQLAVHDAYFAIHPSAGFTTFLTPGAEDGAYRLPDPSYAKDARQ<br>AVAGAAIAMLSKLYMKPKVVPRSPISHNAYAQLQHVLDISVTKAPAACDPA<br>SSSFIFGKAVATAVFDLLFHKEGADQSGYSPKPGPFKFNDEPTHPVELIPVD<br>ANIPDGDKMPRRQYHAPYYGETAKRFGTQTEHMLADPPGIRCAGEVAEY<br>DDAIREVYAMGGAPGLNTTKRTPHQTVQGMFWAYDGPKLIGTPPRLYNQ<br>IVRKIAVTYKKDNDLVNSEVNNADFARLLALVNVAMTDAGIFAWKEKWEF<br>EFWRPLSGVRDDVLRDPEGKASTAAIHSGLASAPQLQNSDEAPFKPPFPAY<br>PSGHATFGAAAFQMVRKYYNGRLGKWATTSRDTIAVEMFVSEELNGVSR<br>DLSNPYDPKRPITDQPGIVPTRMPRRFSSCWEMMFENAVSRIFLGVHWRF<br>DAAAGQDILIPTTKKDVYAVDDKGAALFKNVEDIRYKTKGTRKGHKGLLPIG<br>GVPLGIEIANEIYNNKLSPTPPGEQPMPQPPQHQGPPRKKGELAEAKDEE<br>QAPMMDVAP
SlVCPO	*Stemphylium lycopersici*	MDPVTPIPLPKIEEPEDYNTNYILYWHHVGLEHNRVTHTVGGPQTGPPISA<br>RALGMLQLAAHDAYFAINPSCDFTTFLTPGAENAAYRLPDLNGANDARQA<br>VAGASIKMLSELYSKPVTQPNPNPGANISDNAYEQLELVLDRSIADAPGGV<br>DQASASFVFGQAVAEVFFNLLFHPPGASQEGYHPTPGRYKFDDEPTHPVV

		LIPVNPNNPDGPKMPFRQYHGPFYGTSAKRLGTQTEHMIADPPGIRSAAD<br>EGAEYDDSIRVAIAMGGAIGLNSTKRSPYQTTQGIYWAYDGSNLIGTPPRF<br>YNQIVRRIAVTYKKESDLAASETNNADFARLLALVNVACADAGIFSWKEKW<br>EFEFWRPLSGVRDDGRPDHGDPFWLTLGAPATNTNDIPFKPPFPAYPSGH<br>ATFGGAAFQMVRRYYNGRVGTWKNDEPDTIAIDMMVSEELNGLSRDLR<br>QPYDPSAPITDQPGIVRTRVVRHFSSAWELMFENAISRLFLGVHWRFDAA<br>AARDILIPTTTKDVYAKDENGATVFQNVEDVRYTTLGTREGHEGLLPIGGVP<br>LGIGIADEIFESGLRPTPVERQPMGDGGEGVGGRKEEVMVVDGGMWEG<br>EQVPAMDQAP
CcVCPO	*Curvularia clavata*	MGSVTPIPLPKIDEPEEYNTNYILFWNHVGLELNRVTHTVGGPLTGPPLSAR<br>ALGMLHLAIHDAYFSIYPPTNFTTFLSPDAENAAYRLPSPNGANDARQAVA<br>GAALKMLTSLYMKPVEEPNPNPGANISDNAYAQLALVIDRSVLKAPGGVD<br>RESASFMFGEAVADVFFALLNDPRGASQEGYHPTPGRYKFDDEPTHPVVLI<br>PVDPNNPNGPKKPFRQYHAPFYGKTTKRFATQSEHFLADPPGLRSNADET<br>AEYDDSIRVAIAMGGAQALNSTKRSPWQTAQGLFWAYDGSNLIGTPPRFY<br>NQIVRRIAVTYKKEEDLANSEVNNADFARLFALVDVACTDAGIFSWKEKWE<br>YEFWRPLSGVRDDGRPDHGDPFWLTLGAPATNTNDIPFKPPFPAYPSGHA<br>TFGGAVFQMVRRYYNGRVGTWKDDEPDNIAIDMVISEELNGLNRDLRQP<br>YDPTAPIEDQPGIVRTRIVRHFDSAWELMFENAISRIFLGVHWRFDAAAAR<br>DILIPTTTKDVYAVDNNGATVFQNVEDIRYTTKGTREDREGLFPIGGVPLGIE<br>IADEIFNNGLRPTPPELQPMPQQTPVQKPVGQQTIKGAWSEEEAPVVKEA<br>P
EtVCPO	*Exserohilum turcica Et28A*	MDPVTPIPLPQIDEPEEYNTNYILYWHHIGLELNRLTHTVGGPQTGPPISAR<br>ALGMLQLAAHDAYFAINPSAEFSTFLTPGAENAEYRLPDLNGADDARQAV<br>AGASLKMLTELYMKPVVQSNPNPGANISDRAYAQLALFIEQSSAEAPGGV<br>DRASASFLFGEAVADVFFKLLFHPPGASQDGYHPTPGRYRFDDEPTHPVVL<br>IPVDPNNPNGPKKPFRQYHGPFYGTTAKRFATQTEHILADPPGLRSAANET<br>AEYDDSIRVAIAMGGAPGLNSTKRSPYQTAQGIFWAYDGSNLIGTPPRLYN<br>QIVRRIAVTYKKEDDLANSEVNNADFARLLALVNVACADAGIFSWKEKWEF

		EFWRPLSGVRDDGRPDHGDPFWLTLGAPATNTNDIPFKPPFPAYPSGHAT<br>FGGAVFQMVRRYYNGRVGTWKDDEPDNIAIDMMVSEELNGLSRDLRQP<br>YDPTAPITDQPGIVRTRIERHFNSAWEMMLENAISRIFLGVHWRFDAAAA<br>RDILIPTVTKDVYAIDNNGASVFQNAEDVRYSTRGTREGHDGLFPIGGVPL<br>GIEIADEIFNAGLKPTPPEAQPMPQPPYVQKPPYGKESQSTMWEEEHAQA<br>MDEAP
AnVBPO (1QI9)	*Ascophyllum nodosum*	QTCSTSDDADDPTPPNERDDEAFASRVAAAKRELEGTGTVCQINNGETDL<br>AAKFHKSLPHDDLGQVDADAFAALEDCILNGDLSICEDVPVGNSEGDPVG<br>RLVNPTAAFAIDISGPAFSATTIPPVPTLPSPELAAQLAEVYWMALARDVPF<br>MQYGTDDITVTAAANLAGMEGFPNLDAVSIGSDGTVDPLSQLFRATFVGV<br>ETGPFISQLLVNSFTIDSITVEPKQETFAPDVNYMVDFDEWLNIQNGGPPA<br>GPELLDDELRFVRNARDLARVTFTDNINTEAYRGALILLGLDAFNRAGVNG<br>PFIDIDRQAGFVNFGISHYFRLIGAAELAQRSSWYQKWQVHRFARPEALG<br>GTLHLTIKGELNADFDLSLLENAELLKRVAAINAAQNPNNEVTYLLPQAIQE<br>GSPTHPSYPSGHATQNGAFATVLKALIGLDRGGDCYPDPVYPDDDGLKLID<br>FRGSCLTFEGEINKLAVNVAFGRQMLGIHYRFDGIQGLLLGETITVRTLHQE<br>LMTFAEESTFEFRLFTGEVIKLFQDGTFTIDGFKCPGLVYTGVENCVS
FdVBPO	*Fucus distichus*	MLCHAADTTRGSPMPDTGVLRLLTSEQRAKGWRRQLEGEKSLGFHPSETP<br>YIKYLEGSETWKKVKLPTDGISASKILGKIMARVRIATALAVVLAAPCLAFDE<br>VTASGVFPEEHKHTGEGRHLQTCTNSDDALDPTAPNRRDNVAFASRRDA<br>ARRERDGTGTVCQITNGETDLATMFHKSLPHDELGQVTADDFAILEDCILN<br>GDFSICEDVPAGDPAGRLVNPTAAFAIDISGPAFSATTIPPVPTLSSPELAAQ<br>LAELYWMALARDVPFMQYGTDEITTTAAANLAGMGGFPNLDAVSIGSDG<br>TVDPFSQLFRATFVGVETGPFVQLLVNSFTIDAITVEPKQETFAPDLNYMVD<br>FDEWLNIQNGGPPAGPEELDEELRFIRNARDLARVSFVDNINTEAYRGSLIL<br>LELGAFSRPGINGPFIDSDRQAGFVNFGTSHYFRLIGAAELAQRASCYQKW<br>QVHRFARPEALGGTLHNTIAGDLDADFDISLLENDELLKRVAEINAAQNPN<br>NEVTYLLPQAIQVGSPTHPSYPSGHATQNGAFATVLKALIGLDRGGECFPN<br>PVFPSDDGLELINFEGACLTYEGEINKLAVNVAFGRQMLGIHYRFDGIQGLL

		LGETITVRTLHQELMTFAEEATFEFRLFTGEVIKLFQDGTFSIDGDMCSGLVYTGVADCQA
SjVBPO	*Saccharina japonica*	MKRVRPTQPRALYSAFSRRHVACALLCVVSCIFVSFNHQTFVGLTALLAAFPPCLGYDEPAEPTQPLLSGSVCRVRDSLDFLDPVPRAKVTLLKRLAIAKDEFSVGPTCHINNGDEENVPLFAGQFHKTLPHDKFGQVDEDAYKKLQECIFTSDINVCDDVPSGAKKNGAKLTNPLGGTAHQVAGADSDNIFIPTPDSLLSERLAAQQSEVYWMALARDIPFGEFATNDLIRLAAENLQGLPAFKGLNIPRSKGGKIDPVTDLFRTTWPGVTTGPVVSQFMLSDFLIDSINVTPKADPLTPFTDYMTSFQPWLDVQNGASDVDTTFDSENPRFIRNGRDLATISFRDLLYTEAFRAALILFTQGALGGSIGPYEEAERQQGFATFGAPHILTAMGSASSSTRHAWYQKWQVHRMLRPEAYGGLVHNTLMKNVITPLPDSILRNTELLNRVEVHNQKMNGDGEKTFLLPMASAQGSPTHPAYPSGHAINVGAYITSLKAFLGFEAGQRCFPDPVVSNNEGTERIPYVPSGREIVGECINDKGKKVDGLTYEGELNKVSANVLIGRSHLGVHWRMDGVYGALVGEVSAVRRLQQELPGLAEARPNDDLKRKEIPPATYNFRLYSGKMLELYGANLYKLDGKLCEGAFTGDDFCDEVQEDDYESFEHIVEDLAKFSLHTEL
EsVBPO	*Ectocarpus siliculosus*	MKAFVGIAALLAASPPCLGATSNEEHQPTHPLLSGSVCRVRDSLDFLSPTERAKVTLQKRVDTAKEEFQVGPTCHITNGDETNVPDFIGQFHKSLPHDKFGTVSKTSYQKLLDCVFSADVNVCDQVPSGASARGAKMINPLGGTAHQVDGADSDNVFITTPDGVLSEELAAQMAEVYWMALTRDIPFSEFATNSLVRAAAENLERLPAFKGLNIPKSAGGKIDPVQDLFRTDWPGVTAGPVVSQLLLSDFAIDNIVVPPKQVTLVKEMDYMTTFQDWLDVQNGASKVKTEFVDEDKPLFIRNGRDLAALAFTDLLYTEAFRAALVMFRQGILSSAEGPYSTSERQVGFATFGEPHILTSLAASSSSTRHAWYAKWQVHRVLRPEAYGGLVHNTLTGRLHTPLPKAILQNTELLNRVRTHNAKCNGDGKGGHGDGTYLLPMAVAEGSPVHPAYPSGHAINLGAYITTLKAFLGFELGQRCYLGDLVVSNDEGTKRIEYVPRKGETCIDQNGREVQGLTYEGELNKVASNVIIGRSHIGVHYRMDGVYGALMGETSAVRRLQQELPNLSEARDTKGSIPPASYKFRLYSGKVLELLAKDIFKLDGRTCKGLYTGDDFCDEHQDGGHYLEHLVNKGGDFAFHVEL

CcVBPO	*Chondrus crispu*	MSDMSTEISPRQQEAYDIRVAAATLALARDRKRKVFEPNGEEDRYFRPNS<br>QILSYLGSYTKGLPHNRSTALVTSPNHFVRFVQAIDSGEPADFIKVPLGPRPA<br>LEFTTEEECIAANLFKSGIATRNDSFDPACLRAWESMGAGLVFDLEGPDAQ<br>AVGMPPAPTLDSDELVAEISELYWMALLRDVRFTDFTNPGPIRRAINTLNR<br>TTWIRAARDPPDSLTPQERARLRGPFNPSSIFRGTLPGDDVGPYLSQFLLVG<br>TEGIGGAQDRADGFISYGAHRIDQRVRHATKELDYMTSWEAFLDVQNGA<br>DVAGRDTFESGSDAFRFITTPRDLATYVHFDALYQAYLNACLILLDNKVKFD<br>QGIPMGEPDFNGNQGIPFQDPDFKDHQRGFAHFGGPHILSLVTEVATRAL<br>KAVRFQKFNTHRRLRPEAVGGLIERFNSNPDDPQFQDVKPLFEALDEDML<br>RRVASHNREQNERSDFGMPRADDFNPAGDTLETMLLPMAFPEGSPMHP<br>SYGAGHATVAGACVTVLKAFFQHDATLDFCYVPSDDGSRLDDASHTLNKK<br>LTVEGELNKVCSNISIGRNWAGVHYFTDYIESILLGEQIALGILEEQMLTFPET<br>FTMTVPLFSGGFRTLISTSDP
HhVBPO	*Halomicronema hongdechloris*	MSFYRIAGRVVSQATGAGLSGLRVEVWDKDLIFDDFLAQATTDAQGAFQ<br>VQFDDTSFRDFIWFDRLPDLYFKVFDGNQLIKSTADSVLWNSRDRNQTVV<br>LPVERAPSLALSPPGARGDGAPPAICLFNPPNIPGARLWDRRLQALEVRRQ<br>AAELARRPAHPEHLSNGEELLYRDTDNQPSHIANYTKGLPHDDRGLLSDAD<br>AYQRFVRGIDSGDTRDFQDTPLGLRTGMASNFPIGSLTWPNARNYRCGP<br>GKVPGAGNTFDLEGPDAQAVTMPPAPTLDSPELLAEMVEVYTMALLRDIP<br>FAKFDSAPEIQQAIDRLNATDWIQSPDALALSPEEESRLRSPFTRQTVFRGIA<br>PGDEVGPYLSQFLLVGNTGLGDAQAISDGYIQYGSIRADQRVRVAAPYRDY<br>MTTWEAWLDVQNGADFRGLELYADDPKFRFMATPRDLATYVHYDALYE<br>AYLNACLIMLSLGVPFDPGIPFQRADHIDKQQGFAHFGGPHILSLVTEVATR<br>ALKAVRFQKFNVHRRARPEAIGGLIHRYASGDRQEFMPVEPLVNGFGESFL<br>EEIADHNRRQNQHSDRSGDASAGSNSYLLPMAFPEGSPMHPAYGAGHG<br>TVAGACVTILKAFFDHGYELPVCYQPSVDGAQLESVTLDRPLTVGAELNKLA<br>ANIAIGRNWAGVHYFTDYIESIRLGEAIALGILEEQKLTYGENFSMTIPLFDG<br>GTIRI

CpVBPO	*Corallina pilulifera*	MGIPADNLQSRAKASFDTRVAAAELALNRGVVPSFANGEELLYRNPDPDN<br>TDPSFIASFTKGLPHDDNGAIIDPDDFLAFVRAINSGDEKEIADLTLGPARDP<br>ETGLPIWRSDLANSLELEVRGWENSSAGLTFDLEGPDAQSIAMPPAPVLTS<br>PELVAEIAELYLMALGREIEFSEFDSPKNAEYIQFAIDQLNGLEWFNTPAKLG<br>DPPAEIRRRRGEVTVGNLFRGILPGSEVGPYLSQYIIVGSKQIGSATVGNKTL<br>VSPNAADEFDGEIAYGSITISQRVRIATPGRDFMTDLKVFLDVQDAADFRG<br>FESYEPGARLIRTIRDLATWVHFDALYEAYLNACLILLANGVPFDPNLPFQQ<br>EDKLDNQDVFVNFGSAHVLSLVTEVATRALKAVRYQKFNIHRRLRPEATGG<br>LISVNKIAPQKGESIFPEVDLAVEELGDILEKAEISNRKQNIADGDPDPDPSFL<br>LPMAFAEGSPFHPSYGSGHAVVAGACVTILKAFFDSGIEIDQVFEVDKDED<br>KLVKSSFKGTLTVAGELNKLADNIAIGRNMAGVHYFSDQFESLLLGEQVAIG<br>ILEEQSLTYGENFFFNLPKFDGTTIQI
LepVBPO	*Leptolyngbya* sp. BC1307	MESRKKRALDLRTAAAKVAYDRAHAQHWANGEELRYRFEDVSNKERAG<br>QPSHLANYTKGLPHDEKDGLICCSADYQQFVRGIDSGDSRDFRDTPLGPKG<br>SETQEQIEWKSHYTQQHKADVRAWESAGAGLLFDLEGPDCQSITMPPAP<br>VLDSDELVAEVAEVYEMALLRDVPFVDFGCNPEVGAAIDRLNKLTWFQNT<br>PLPLTDAAQARRRGQLTAQTAFRGVAVGDTTGPYISQFLLRGTPELGGDR<br>DGVFDGLVAYGAMAFTNKVRTATPYKDYMTTWEWWLDVQNGADLRGK<br>ETYENCDNKYRFITTPRDLATYVHYDALYQAYLNATLALMALKVPFDPGVP<br>FQGRDRQDHQQGFAHFGGPHILSLVTEVATRALKAVRYQKFNVHRRLRPE<br>AIAGRIHQWRETQAPNLAPVSQLSNKISDTLHQVSQHNRVQNEKFLAELS<br>ADGCSDRLKQEFDGENYLLPMAFPEGSPMHPSYGAGHATVAGACITILKA<br>FFDHGYVLPQPYVVSRNGTQLESVQDSTLTLTVEGELNKLAANIAIGRDWA<br>GVHYFTDYYESMRLGEQIALGILEEQKLTYGENFSMTVPLLDGGCARI
CoVBPO	*Corallina officinalis*	MGIPADNLQSRAKASFDTRVSAAELALARGVVPSLANGEELLYRNPDPEN<br>GDPSFIVSFTKGLPHDDNGAIIDPDDFLAFVRAINSGDEKEIADLTLGPARDP<br>DTGLPIWRSDLANSLELEVRGWENSSAGLTFDLEGPDAQSIAMPPAPVLTS<br>PELIAEIAELYLMALGREIEFSEFDSPKNAEYIQFAIDQLNGLEWFNTPAMLG<br>DPPAEIRRRRGEVTVGNLFRGILPGSEVGPYLSQYIIVGSKQIGSATGGNKTL

		VSPNAADEFDGEIAYGSITISQRVRIATPGRDFMTDLKVFLDVQDAADFRG<br>FESYEPGARLIRTIRDLATWVHFDALYEAYLNACLILLANRVPFDPNIPFQQE<br>DKLDNQDVFVNFGDAHVLSLVTEVATRALKAVRYQKFNIHRRLRPEATGG<br>LISVNKIAAEKGESVFPEVDLAVEELEDILEKAEISNRKQNIADGDPDPDPSFL<br>LPQAFAEGSPFHPSYGSGHAVVAGACVTILKAFFDSNFQIDQVFEVDKDED<br>KLVKSSFKGTLTVAGELNKLADNIAIGRNMAGVHYFSDQFESILLGEQVAIGI<br>LEEQSLTYGENFFFNLPKFDGTTIQI

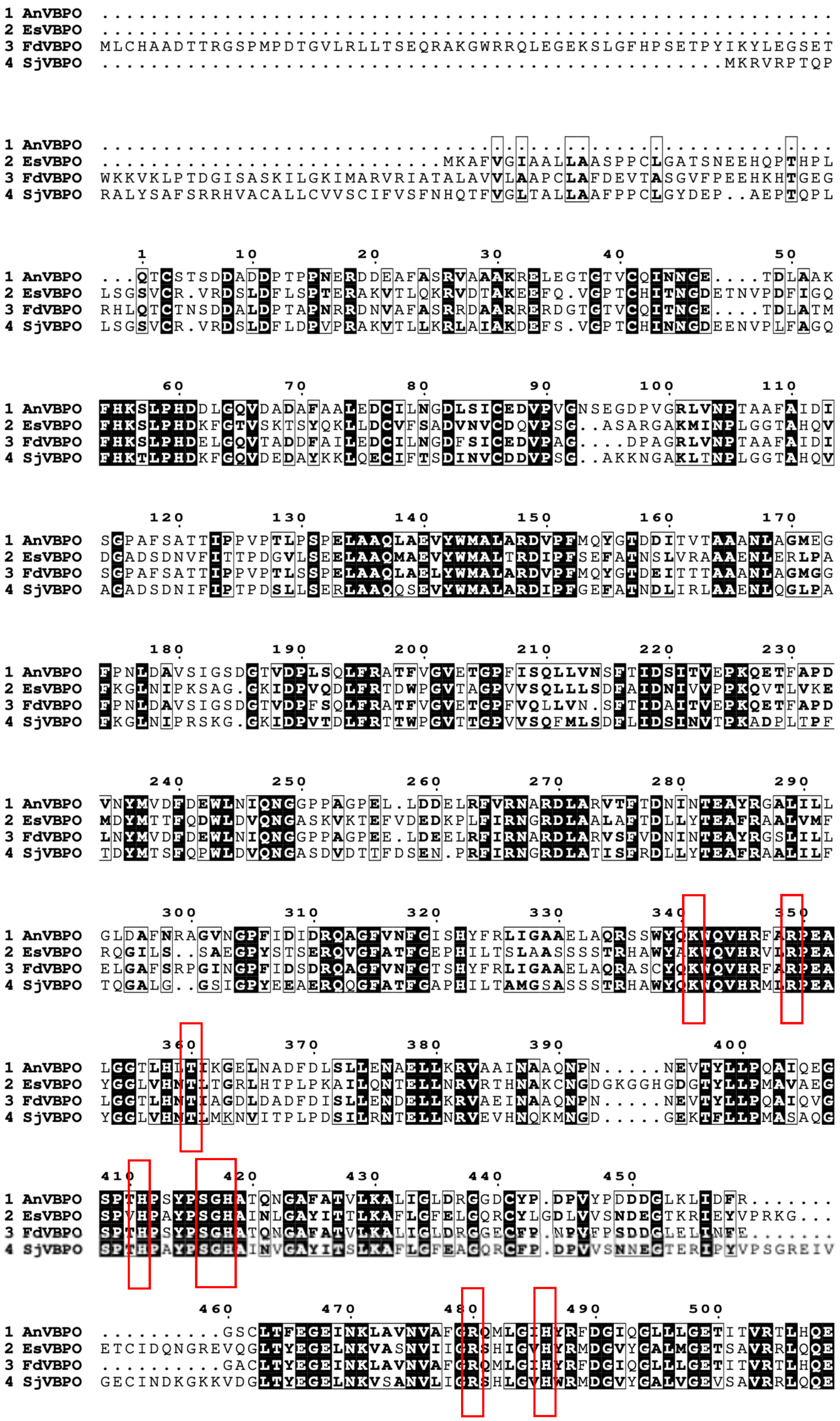
1 AnVBPO ....
2 EsVBPO ....
3 FdVBPO MLCHAADTTRGSPMPDTGVLRLLTSEQRAKGWRRQLEGEKSLGFHPSETPYIKYLEGSET
4 SjVBPO ....MKRVRPTQP
1 AnVBPO ....
2 EsVBPO .............................MKAFVGIAALLAASPPCLGATSNEEHQPTHPL
3 FdVBPO WKKVKLPTDGISASKILGKIMARVRIATALAVVLAAPCLAFDEVTASGVFPEEHKHTGEG
4 SjVBPO RALYSAFSRRHVACALLCVVSCIFVSFNHQTFVGLTALLAAFPPCLGYDEP..AEPTQPL
1 10 20 30 40 50
1 AnVBPO ...QTCSTSDDADDPTPPNERDDEAFASRVAAAKRELEGTGTVCQINNGE....TDLAAK
2 EsVBPO LSGSVCR.VRDSLDFLSPTERAKVTLQKRVDTAKEEFQ.VGPTCHITNGDETNVPDFIGQ
3 FdVBPO RHLQTCTNSDDALDPTAPNRRDNVAFASRRDAARRERDGTGTVCQITNGE....TDLATM
4 SjVBPO LSGSVCR.VRDSLDFLDPVPRAKVTLLKRLAIAKDEFS.VGPTCHINNGDEENVPLFAGQ
60 70 80 90 100 110
1 AnVBPO FHKSLPHDDLGQVDADAFAALEDCILNGDLSICEDVPVGNSEGDPVGRLVNPTAAFAIDI
2 EsVBPO FHKSLPHDKFGTVSKTSYQKLLDCVFSADVNVCDQVPSG..ASARGAKMINPLGGTAHQV
3 FdVBPO FHKSLPHDELGQVTADDFAILEDCILNGDFSICEDVPAG....DPAGRLVNPTAAFAIDI
4 SjVBPO FHKTLPHDKFGQVDEDAYKKLQECIFTSDINVCDDVPSG..AKKNGAKLTNPLGGTAHQV
120 130 140 150 160 170
1 AnVBPO SGPAFSATTIPPVPTLPSPELAAQLAEVYWMALARDVPFMQYGTDDITVTAAANLAGMEG
2 EsVBPO DGADSDNVFITTPDGVLSEELAAQMAEVYWMALTRDIPFSEFATNSLVRAAAENLERLPA
3 FdVBPO SGPAFSATTIPPVPTLSSPELAAQLAELYWMALARDVPFMQYGTDEITTTAAANLAGMGG
4 SjVBPO AGADSDNIFIPTPDSLLSERLAAQQSEVYWMALARDIPFGEFATNDLIRLAAENLQGLPA
180 190 200 210 220 230
1 AnVBPO FPNLDAVSIGSDGTVDPLSQLFRATFVGVETGPFISQLLVNSFTIDSITVEPKQETFAPD
2 EsVBPO FKGLNIPKSAG.GKIDPVQDLFRTDWPGVTAGPVVSQLLLSDFAIDNIVVPPKQVTLVKE
3 FdVBPO FPNLDAVSIGSDGTVDPFSQLFRATFVGVETGPFVQLLVN.SFTIDAITVEPKQETFAPD
4 SjVBPO FKGLNIPRSKG.GKIDPVTDLFRTTWPGVTTGPVVSQFMLSDFLIDSINVTPKADPLTPF
240 250 260 270 280 290
1 AnVBPO VNYMVDFDEWLNIQNGGPPAGPEL.LDDELRFVRNARDLARVTFTDNINTEAYRGALILL
2 EsVBPO MDYMTTFQDWLDVQNGASKVKTEFVDEDKPLFIRNGRDLAALAFTDLLYTEAFRAALVMF
3 FdVBPO LNYMVDFDEWLNIQNGGPPAGPEE.LDEELRFIRNARDLARVSFVDNINTEAYRGSLILL
4 SjVBPO TDYMTSFQPWLDVQNGASDVDTTFDSEN.PRFIRNGRDLATISFRDLLYTEAFRAALILF
300 310 320 330 340 350
1 AnVBPO GLDAFNRAGVNGPFIDIDRQAGFVNFGISHYFRLIGAAELAQRSSWYQKWQVHRFARPEA
2 EsVBPO RQGILS..SAEGPYSTSERQVGFATFGEPHILTSLAASSSSTRHAWYAKWQVHRVLRPEA
3 FdVBPO ELGAFSRPGINGPFIDSDRQAGFVNFGTSHYFRLIGAAELAQRASCYQKWQVHRFARPEA
4 SjVBPO TQGALG..GSIGPYEEAERQQGFATFGAPHILTAMGSASSSTRHAWYQKWQVHRMLRPEA
360 370 380 390 400
1 AnVBPO LGGTLHLTIKGELNADFDLSLLENAELLKRVAAINAAQNPN.....NEVTYLLPQAIQEG
2 EsVBPO YGGLVHNTLTGRLHTPLPKAILQNTELLNRVRTHNAKCNGDGKGGHGDGTYLLPMAVAEG
3 FdVBPO LGGTLHNTIAGDLDADFDISLLENDELLKRVAEINAAQNPN.....NEVTYLLPQAIQVG
4 SjVBPO YGGLVHNTLMKNVITPLPDSILRNTELLNRVEVHNQKMNGD.....GEKTFLLPMASAQG
410 420 430 440 450
1 AnVBPO SPTHPSYPSGHATQNGAFATVLKALIGLDRGGDCYP.DPVYPDDDGLKLIDFR.......
2 EsVBPO SPVHPAYPSGHAINLGAYITTLKAFLGFELGQRCYLGDLVVSNDEGTKRIEYVPRKG...
3 FdVBPO SPTHPSYPSGHATQNGAFATVLKALIGLDRGGECFP.NPVFPSDDGLELINFE.......
4 SjVBPO SPTHPAYPSGHAINVGAYITSLKAFLGFEAGQRCFP.DPVVSNNEGTERIPYVPSGREIV
460 470 480 490 500
1 AnVBPO ..........GSCLTFEGEINKLAVNVAFGRQMLGIHYRFDGIQGLLLGETITVRTLHQE
2 EsVBPO ETCIDQNGREVQGLTYEGELNKVASNVIIGRSHIGVHYRMDGVYGALMGETSAVRRLQQE
3 FdVBPO ..........GACLTYEGEINKLAVNVAFGRQMLGIHYRFDGIQGLLLGETITVRTLHQE
4 SjVBPO GECINDKGKKVDGLTYEGELNKVSANVLIGRSHLGVHWRMDGVYGALVGEVSAVRRLQQE

```
              510       520       530       540       550
1 AnVBPO  LMTFAEES.............TFEFRLFTGEVIKLFQDGTFTIDGFKCPGLVYTGVENCV
2 EsVBPO  LPNLSEAR....DTKGSIPPASYKFRLYSGKVLELLAKDIFKLDGRTCKG.LYTGDDFCD
3 FdVBPO  LMTFAEEA.............TFEFRLFTGEVIKLFQDGTFSIDGDMCSGLVYTGVADCQ
4 SjVBPO  LPGLAEARPNDDLKRKEIPPATYNFRLYSGKMLELYGANLYKLDGKLCEG.AFTGDDFCD

1 AnVBPO  S.........................
2 EsVBPO  EHQ.DGGHYLEHLVNKGGDFAFHVEL
3 FdVBPO  A.........................
4 SjVBPO  EVQEDDYESFEHIVEDLAKFSLHTEL
```

Figure S1. Sequence Alignments of *An*VBPO with putative VHPOs (Amino acids marked in red are the binding sites and active sites provided by uniprot).

```
1 CpVBPO     ............................................................
2 CoVBPO     ............................................................
3 HhVBPO     MSFYRIAGRVVSQATGAGLSGLRVEVWDKDLIFDDFLAQATTDAQGAFQVQFDDTSFRDF
4 CcVBPO     ............................................................
5 LepVBPO    ............................................................

1 CpVBPO     ............................................................
2 CoVBPO     ............................................................
3 HhVBPO     IWFDRLPDLYFKVFDGNQLIKSTADSVLWNSRDRNQTVVLPVERAPSLALSPPGARGDGA
4 CcVBPO     ............................................................
5 LepVBPO    ............................................................

                      1        10        20        30        40        50
1 CpVBPO     .........MGIPADNLQSRAKASFDTRVAAAELALNRGVVPSFANGEELLYRNPDPDN.
2 CoVBPO     .........MGIPADNLQSRAKASFDTRVSAAELALARGVVPSLANGEELLYRNPDPEN.
3 HhVBPO     PPAICLFNPPNIPGARLWDRRLQALEVRRQAAELARRPAHPEHLSNGEELLYRDTDN...
4 CcVBPO     .........MSDMSTEISPRQQEAYDIRVAAATLALARDRKRKVFEPNGEEDRYFRPNS.
5 LepVBPO    ................MESRKKRALDLRTAAAKVAYDRAHAQHWANGEELRYRFEDVSNK

                        60        70        80        90       100
1 CpVBPO     ...TDPSFIASFTKGLPHDDN.GAIIDPDDFLAFVRAINSGDEKEIADLTLGPA......
2 CoVBPO     ...GDPSFIVSFTKGLPHDDN.GAIIDPDDFLAFVRAINSGDEKEIADLTLGPA......
3 HhVBPO     ....QPSHIANYTKGLPHDDR.GLLSDADAYQRFVRGIDSGDTRDFQDTPLGLR......
4 CcVBPO     ...QILSYLGSYTKGLPHNRSTALVTSPNHFVRFVQAIDSGEPADFIKVPLGPRPALEFT
5 LepVBPO    ERAGQPSHLANYTKGLPHDEKDGLICCSADYQQFVRGIDSGDSRDFRDTPLGPK......

                        110       120       130       140       150
1 CpVBPO     .....RDPETGLPIWRSDLANSLELEVRGWENSSAGLTFDLEGPDAQSIAMPPAPVLTSP
2 CoVBPO     .....RDPDTGLPIWRSDLANSLELEVRGWENSSAGLTFDLEGPDAQSIAMPPAPVLTSP
3 HhVBPO     .....TGMASNFPIGSLTWPNARNYRCGPGKVPGAGNTFDLEGPDAQAVTMPPAPTLDSP
4 CcVBPO     TEEECIAANLFKSGIATRNDSFDPACLRAWESMGAGLVFDLEGPDAQAVGMPPAPTLDSD
5 LepVBPO    .....GSETQEQIEWKSHYTQQHKADVRAWESAGAGLLFDLEGPDCQSITMPPAPVLDSD

                 160       170       180       190       200       210
1 CpVBPO     ELVAEIAELYLMALGREIEFSEFDSPKNAEYIQFAIDQLNGLEWFNTPAKLGD..PPAEI
2 CoVBPO     ELIAEIAELYLMALGREIEFSEFDSPKNAEYIQFAIDQLNGLEWFNTPAMLGD..PPAEI
3 HhVBPO     ELLAEMVEVYTMALLRDIPFAKFD...SAPEIQQAIDRLNATDWIQSPDALAL..SPEEE
4 CcVBPO     ELVAEISELYWMALLRDVRFTDFT...NPGPIRRAINTLNRTTWIRAARDPPDSLTPQER
5 LepVBPO    ELVAEVAEVYEMALLRDVPFVDFG...CNPEVGAAIDRLNKLTWFQN.TPLPL..TDAAQ

                  220       230       240       250       260       270
1 CpVBPO     RRRRGEVTVGNLFRGILPGSEVGPYLSQYIIVGSKQIGSATVGNKTLVSPNAADEFDGEI
2 CoVBPO     RRRRGEVTVGNLFRGILPGSEVGPYLSQYIIVGSKQIGSATGGNKTLVSPNAADEFDGEI
3 HhVBPO     SRLRSPFTRQTVFRGIAPGDEVGPYLSQFLLVG...........NTGLG.DAQAISDGYI
4 CcVBPO     ARLRGPFNPSSIFRGTLPGDDVGPYLSQFLLVG............TEGIGGAQDRADGFI
5 LepVBPO    ARRRGQLTAQTAFRGVAVGDTTGPYISQFLLRG...........TPELGGDRDGVFDGLV

                  280       290       300       310       320       330
1 CpVBPO     AYGSITISQRVRIATPGRDFMTDLKVFLDVQDAADFRG...FESYEPGARLIRTIRDLAT
2 CoVBPO     AYGSITISQRVRIATPGRDFMTDLKVFLDVQDAADFRG...FESYEPGARLIRTIRDLAT
3 HhVBPO     QYGSIRADQRVRVAAPYRDYMTTWEAWLDVQNGADFRGLELYADDP.KFRFMATPRDLAT
4 CcVBPO     SYGAHRIDQRVRHATKELDYMTSWEAFLDVQNGADVAGRDTFESGSDAFRFITTPRDLAT
5 LepVBPO    AYGAMAFTNKVRTATPYKDYMTTWEWWLDVQNGADLRGKETYENCDNKYRFITTPRDLAT
```

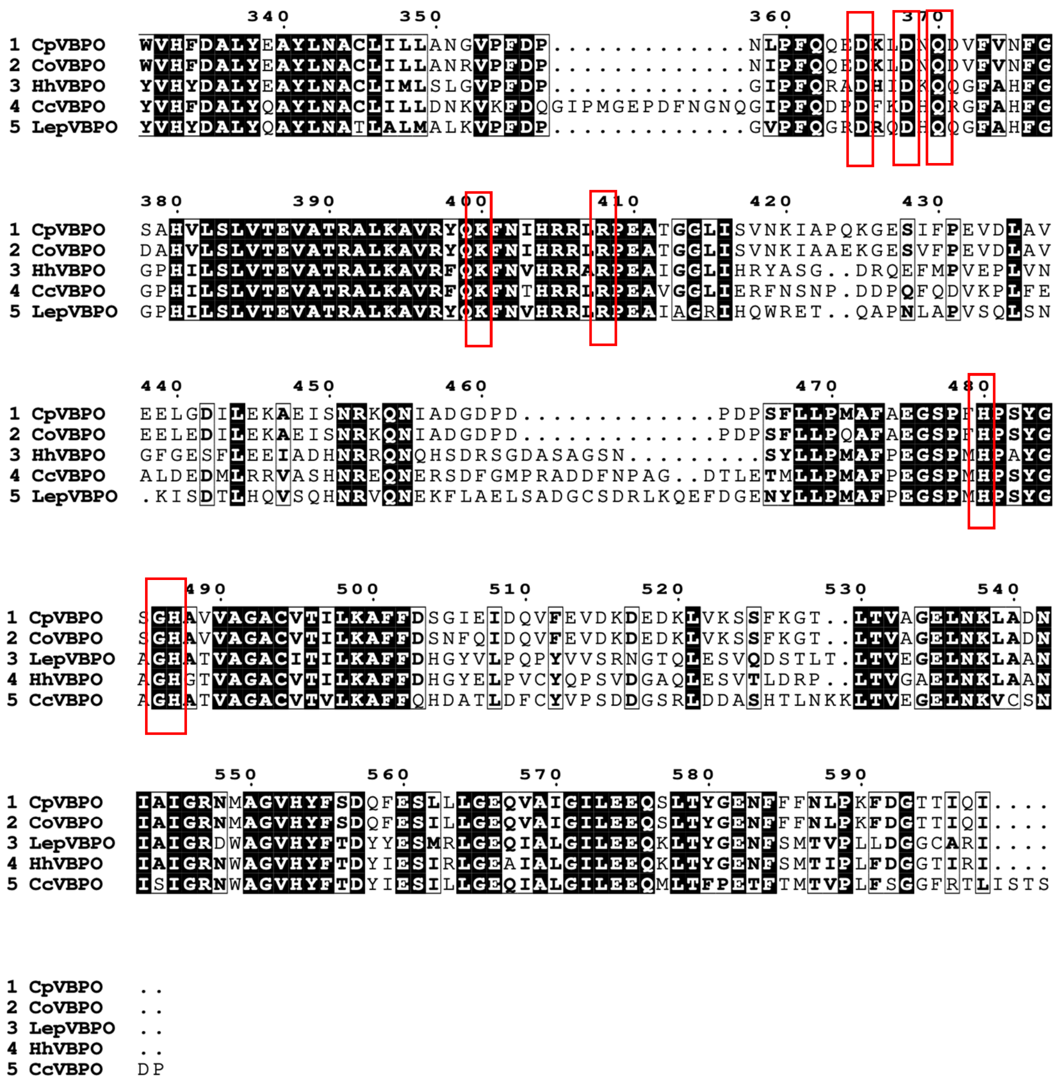

Figure S2. Sequence Alignments of *Cp*VBPO with putative VHPOs (Amino acids marked in red are the binding sites and active sites provided by uniprot).

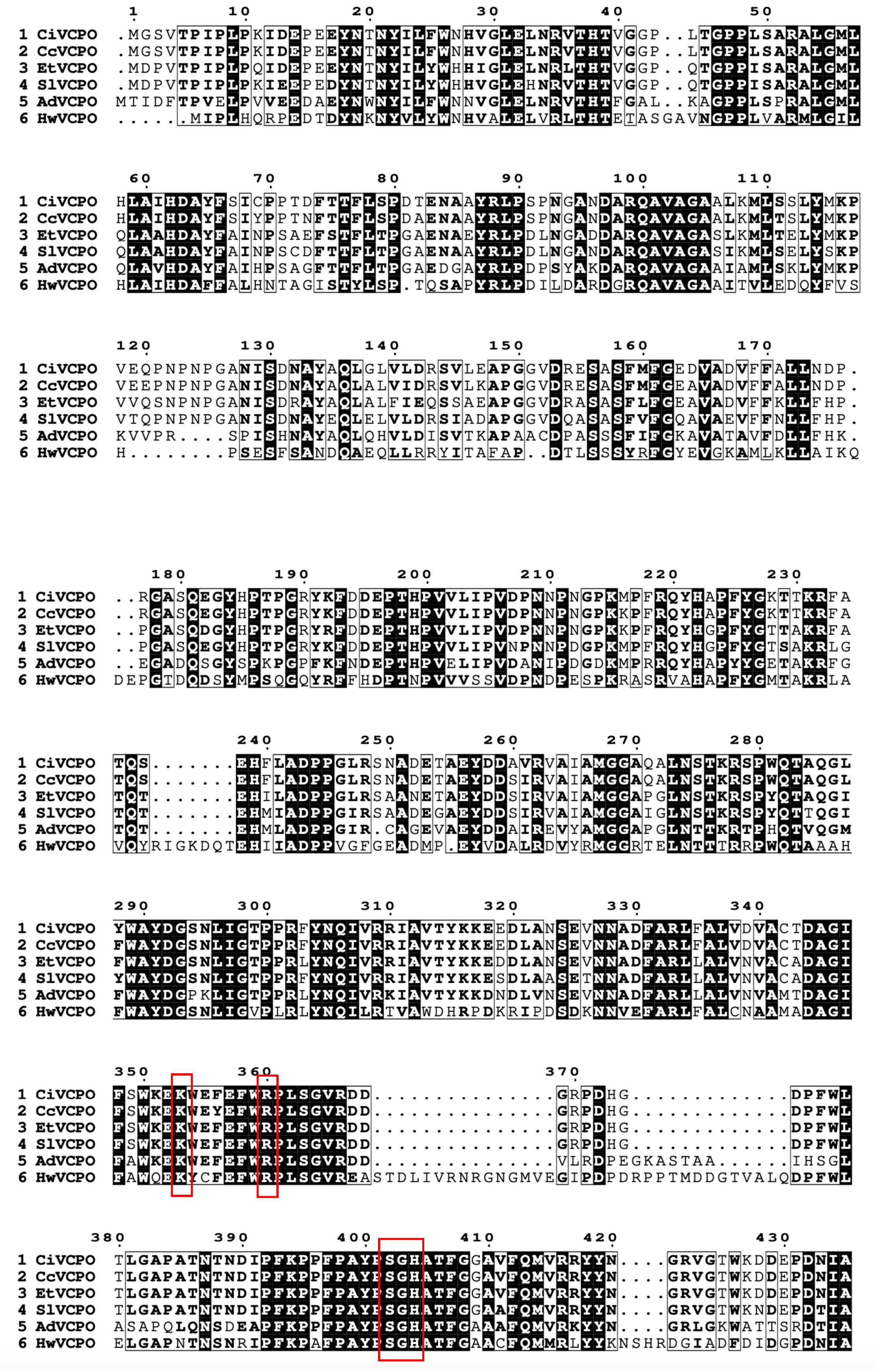
1 10 20 30 40 50
1 CiVCPO .MGSVTPIPLPKIDEPEEYNTNYILFWNHVGLELNRVTHTVGGP..LTGPPLSARALGML
2 CcVCPO .MGSVTPIPLPKIDEPEEYNTNYILFWNHVGLELNRVTHTVGGP..LTGPPLSARALGML
3 EtVCPO .MDPVTPIPLPQIDEPEEYNTNYILYWHHIGLELNRLTHTVGGP..QTGPPISARALGML
4 SlVCPO .MDPVTPIPLPKIEEPEDYNTNYILYWHHVGLEHNRVTHTVGGP..QTGPPISARALGML
5 AdVCPO MTIDFTPVELPVVEEDAEYNWNYILFWNNVGLELNRVTHTFGAL..KAGPPLSPRALGML
6 HwVCPO .....MIPLHQRPEDTDYNKNYVLYWNHVALELVRLTHTETASGAVNGPPLVARMLGIL
60 70 80 90 100 110
1 CiVCPO HLAIHDAYFSICPPTDFTTFLSPDTENAAYRLPSPNGANDARQAVAGAALKMLSSLYMKP
2 CcVCPO HLAIHDAYFSIYPPTNFTTFLSPDAENAAYRLPSPNGANDARQAVAGAALKMLTSLYMKP
3 EtVCPO QLAAHDAYFAINPSAEFSTFLTPGAENAEYRLPDLNGADDARQAVAGASLKMLTELYMKP
4 SlVCPO QLAAHDAYFAINPSCDFTTFLTPGAENAAYRLPDLNGANDARQAVAGASIKMLSELYSKP
5 AdVCPO QLAVHDAYFAIHPSAGFTTFLTPGAEDGAYRLPDPSYAKDARQAVAGAAIAMLSKLYMKP
6 HwVCPO HLAIHDAFFALHNTAGISTYLSP.TQSAPYRLPDILDARDGRQAVAGAAITVLEDQYFVS
120 130 140 150 160 170
1 CiVCPO VEQPNPNPGANISDNAYAQLGLVLDRSVLEAPGGVDRESASFMFGEDVADVFFALLNDP.
2 CcVCPO VEEPNPNPGANISDNAYAQLALVIDRSVLKAPGGVDRESASFMFGEAVADVFFALLNDP.
3 EtVCPO VVQSNPNPGANISDRAYAQLALFIEQSSAEAPGGVDRASASFLFGEAVADVFFKLLFHP.
4 SlVCPO VTQPNPNPGANISDNAYEQLELVLDRSIADAPGGVDQASASFVFGQAVAEVFFNLLFHP.
5 AdVCPO KVVPR....SPISHNAYAQLQHVLDISVTKAPAACDPASSSFIFGKAVATAVFDLLFHK.
6 HwVCPO H........PSESFSANDQAEQLLRRYITAFAP..DTLSSSYRFGYEVGKAMLKLLAIKQ
180 190 200 210 220 230
1 CiVCPO ..RGASQEGYHPTPGRYKFDDEPTHPVVLIPVDPNNPNGPKMPFRQYHAPFYGKTTKRFA
2 CcVCPO ..RGASQEGYHPTPGRYKFDDEPTHPVVLIPVDPNNPNGPKKPFRQYHAPFYGKTTKRFA
3 EtVCPO ..PGASQDGYHPTPGRYRFDDEPTHPVVLIPVDPNNPNGPKKPFRQYHGPFYGTTAKRFA
4 SlVCPO ..PGASQEGYHPTPGRYKFDDEPTHPVVLIPVNPNNPDGPKMPFRQYHGPFYGTSAKRLG
5 AdVCPO ..EGADQSGYSPKPGPFKFNDEPTHPVELIPVDANIPDGDKMPRRQYHAPYYGETAKRFG
6 HwVCPO DEPGTDQDSYMPSQGQYRFFHDPTNPVVVSSVDPNDPESPKRASRVAHAPFYGMTAKRLA
240 250 260 270 280
1 CiVCPO TQS.......EHFLADPPGLRSNADETAEYDDAVRVAIAMGGAQALNSTKRSPWQTAQGL
2 CcVCPO TQS.......EHFLADPPGLRSNADETAEYDDSIRVAIAMGGAQALNSTKRSPWQTAQGL
3 EtVCPO TQT.......EHILADPPGLRSAANETAEYDDSIRVAIAMGGAPGLNSTKRSPYQTAQGI
4 SlVCPO TQT.......EHMIADPPGIRSAADEGAEYDDSIRVAIAMGGAIGLNSTKRSPYQTTQGI
5 AdVCPO TQT.......EHMLADPPGIR.CAGEVAEYDDAIREVYAMGGAPGLNTTKRTPHQTVQGM
6 HwVCPO VQYRIGKDQTEHIIADPPVGFGEADMP.EYVDALRDVYRMGGRTELNTTTRRPWQTAAAH
290 300 310 320 330 340
1 CiVCPO YWAYDGSNLIGTPPRFYNQIVRRIAVTYKKEEDLANSEVNNADFARLFALVDVACTDAGI
2 CcVCPO FWAYDGSNLIGTPPRFYNQIVRRIAVTYKKEEDLANSEVNNADFARLFALVDVACTDAGI
3 EtVCPO FWAYDGSNLIGTPPRLYNQIVRRIAVTYKKEDDLANSEVNNADFARLLALVNVACADAGI
4 SlVCPO YWAYDGSNLIGTPPRFYNQIVRRIAVTYKKESDLAASETNNADFARLLALVNVACADAGI
5 AdVCPO FWAYDGPKLIGTPPRLYNQIVRKIAVTYKKDNDLVNSEVNNADFARLLALVNVAMTDAGI
6 HwVCPO FWAYDGSNLIGVPLRLYNQILRTVAWDHRPDKRIPDSDKNNVEFARLFALCNAAMADAGI
350 360 370
1 CiVCPO FSWKEKWEFEFWRPLSGVRDD...............GRPDHG.............DPFWL
2 CcVCPO FSWKEKWEYEFWRPLSGVRDD...............GRPDHG.............DPFWL
3 EtVCPO FSWKEKWEFEFWRPLSGVRDD...............GRPDHG.............DPFWL
4 SlVCPO FSWKEKWEFEFWRPLSGVRDD...............GRPDHG.............DPFWL
5 AdVCPO FAWKEKWEFEFWRPLSGVRDD...............VLRDPEGKASTAA......IHSGL
6 HwVCPO FAWQEKYCFEFWRPLSGVREASTDLIVRNRGNGMVEGIPDPDRPPTMDDGTVALQDPFWL
380 390 400 410 420 430
1 CiVCPO TLGAPATNTNDIPFKPPFPAYPSGHATFGGAVFQMVRRYYN....GRVGTWKDDEPDNIA
2 CcVCPO TLGAPATNTNDIPFKPPFPAYPSGHATFGGAVFQMVRRYYN....GRVGTWKDDEPDNIA
3 EtVCPO TLGAPATNTNDIPFKPPFPAYPSGHATFGGAVFQMVRRYYN....GRVGTWKDDEPDNIA
4 SlVCPO TLGAPATNTNDIPFKPPFPAYPSGHATFGGAAFQMVRRYYN....GRVGTWKNDEPDTIA
5 AdVCPO ASAPQLQNSDEAPFKPPFPAYPSGHATFGAAAFQMVRKYYN....GRLGKWATTSRDTIA
6 HwVCPO ELGAPNTNSNRIPFKPAFPAYPSGHATFGAACFQMMRLYYKNSHRDGIAIDFDIDGPDNIA

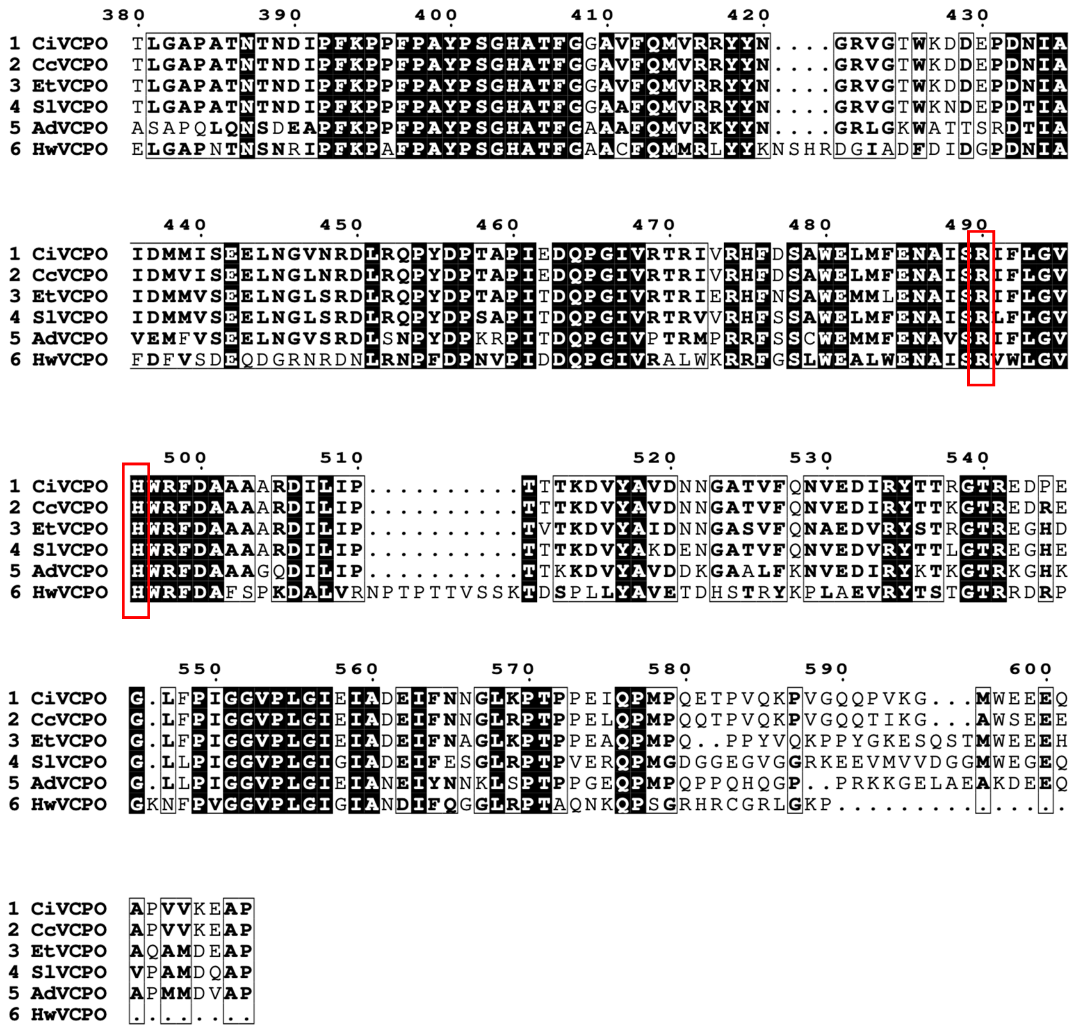

Figure S3. Sequence Alignments of *Ci*VCPO with putative VHPOs (Amino acids marked in red are the binding sites and active sites provided by uniprot).

Table S2. Molecular docking results of MPS molecular with *An*VBPO and *Cp*VBPO

Protein	Binding Energy (kcal/mol)	Number in Cluster	Intermolecular Energy (kcal/mol)	Electrostatic Energy (kcal/mol)	Internal Energy (kcal/mol)
*An*VBPO	-3.32	174	-3.59	-0.18	-0.04
*Cp*VBPO	-3.71	180	-3.98	-0.09	-0.04

GC chromatograms

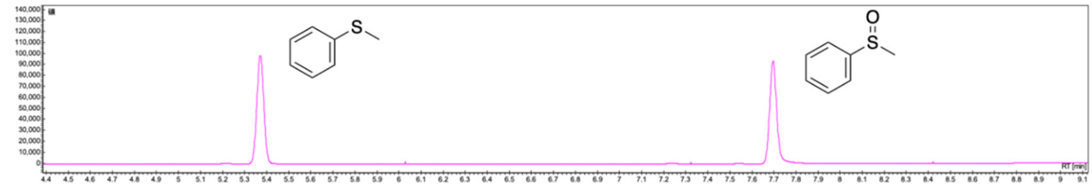

Figure S4. Representative GC chromatogram of **1a** and **1b.**

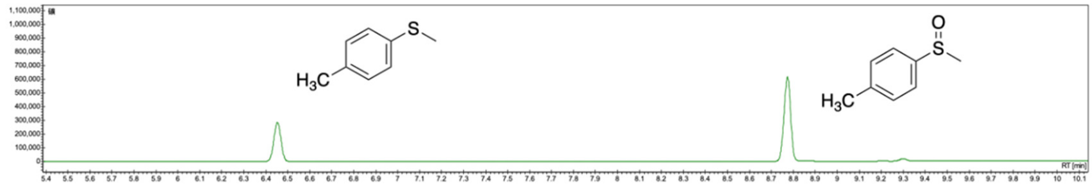

Figure S5. Representative GC chromatogram of **2a** and **2b**.

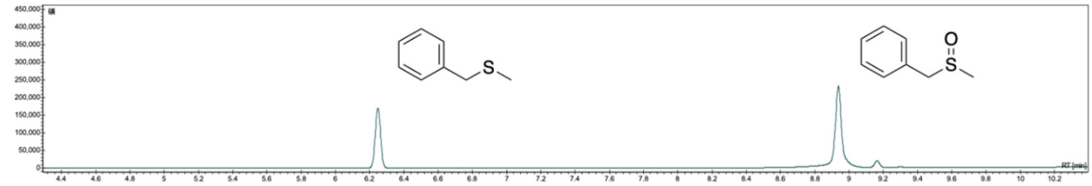

Figure S6. Representative GC chromatogram of **3a** and **3b**.

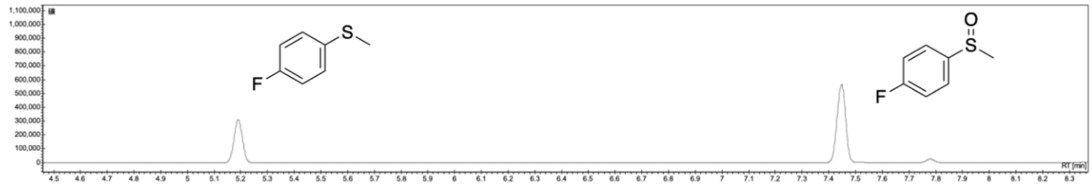

Figure S7. Representative GC chromatogram of **4a** and **4b**.

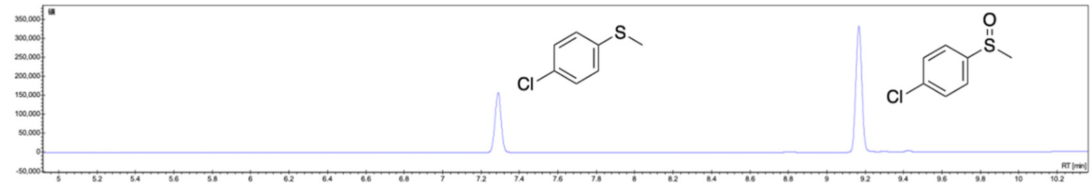

Figure S8. Representative GC chromatogram of **5a** and **5b**.

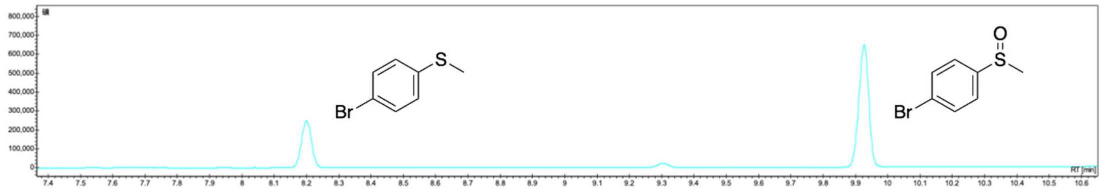

Figure S9. Representative GC chromatogram of **6a** and **6b**.

HPLC chromatograms

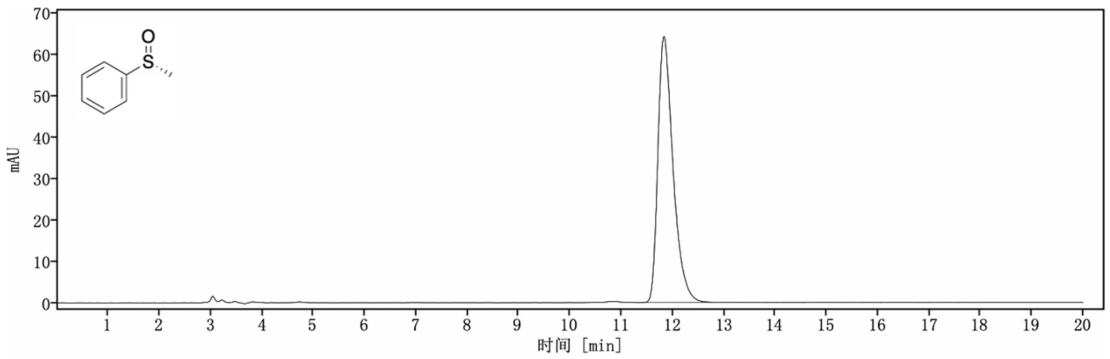

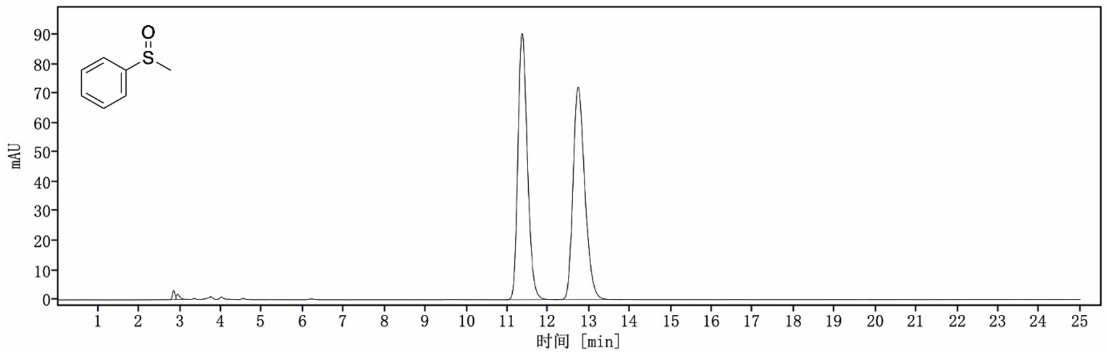

Figure S10. Representative chiral HPLC chromatogram of **1b**

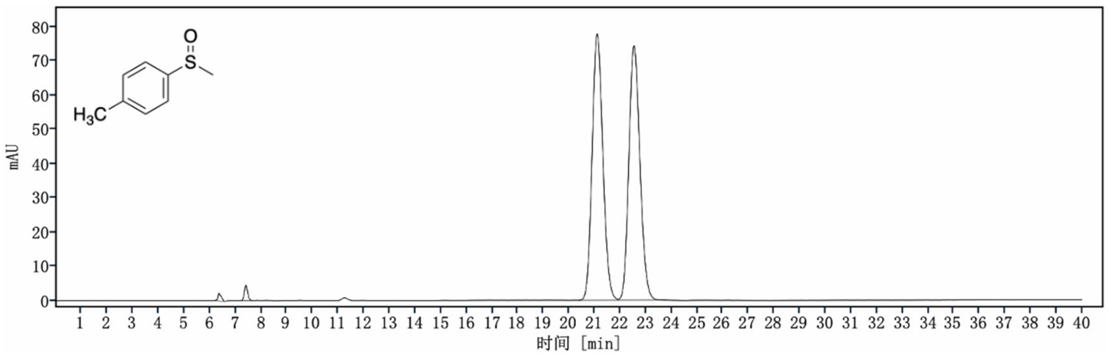

Figure S11. Representative chiral HPLC chromatogram of **2b**

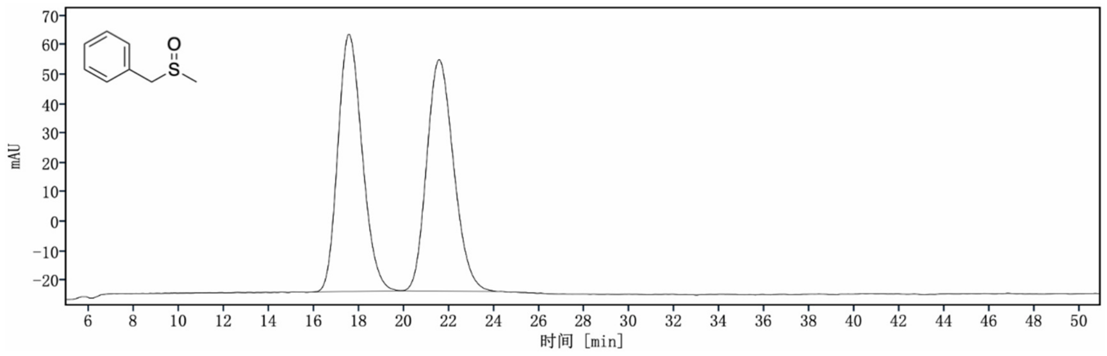

Figure S12. Representative chiral HPLC chromatogram of **3b**

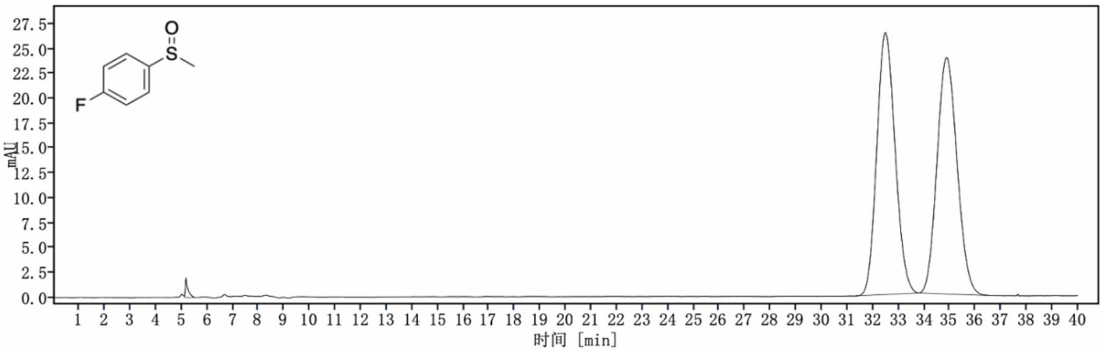

Figure S13. Representative chiral HPLC chromatogram of **4b**

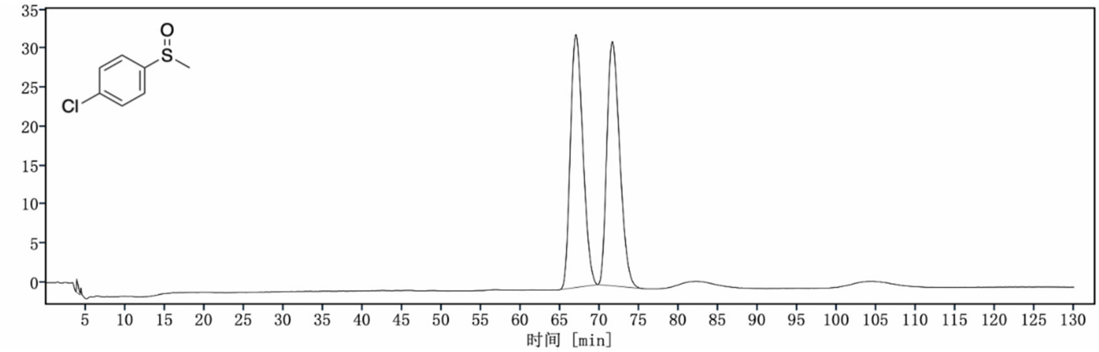

Figure S14. Representative chiral HPLC chromatogram of **5b**

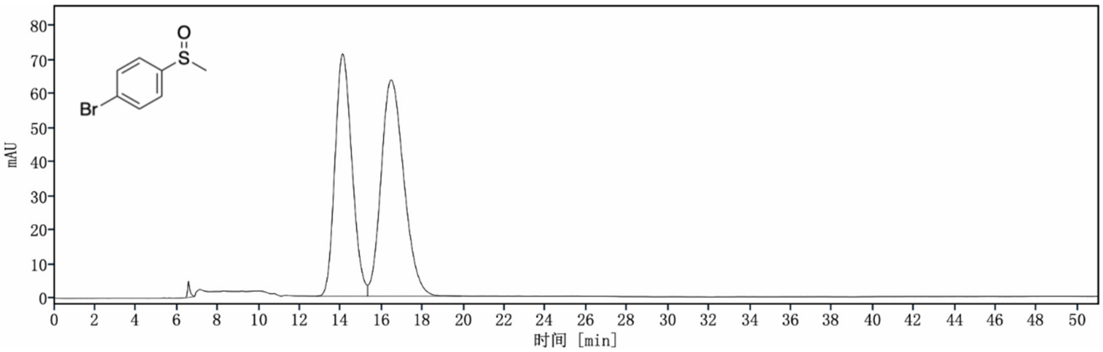

Figure S15. Representative chiral HPLC chromatogram of **6b**

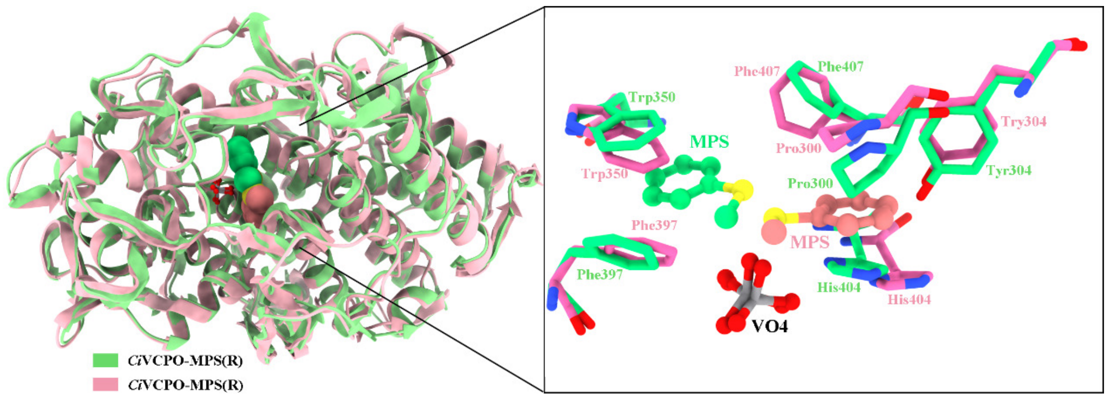

Figure S16. The analysis of MPS molecule binding to *Ci*VCPO active site, which is clear that MPS can bind to the left and right sides of the VO_4^{3-} moiety in the substrate-binding pockets of *Ci*VCPO, respectively, which leading to the poor enantioselectivities.

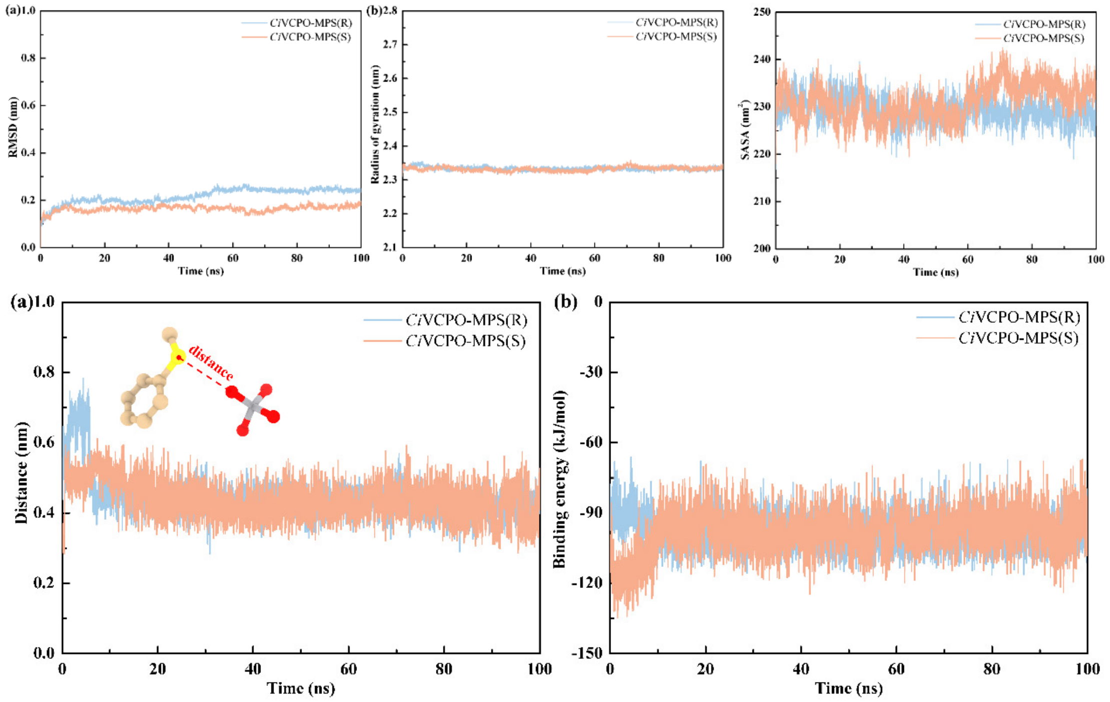

Figure S17. Molecular dynamics (MD) simulations analysis. Backbone RMSD (a); Backbone Rg (b); Total SASA (c); The change in catalytic atomic distance between MPS and VO_4^{3-} (d); and the change in binding energy between MPS and the protein (e) versus simulation time..

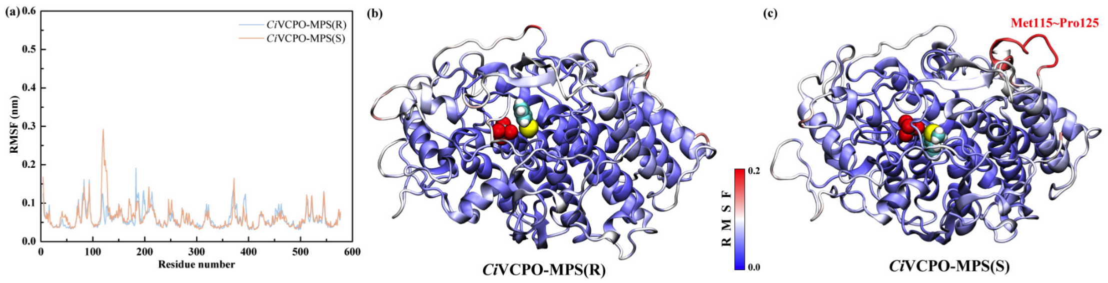

Figure S18. The distribution of root mean square fluctuation (RMSF) for both systems (a and c) and the cor-responding regions exhibiting notable alterations in protein structure (b and d).
